# Synthesis of alpha-tetrasubstituted triazoles by copper-catalyzed silyl deprotection/azide cycloaddition

**DOI:** 10.3762/bjoc.11.154

**Published:** 2015-08-14

**Authors:** Zachary L Palchak, Paula T Nguyen, Catharine H Larsen

**Affiliations:** 1 Department of Chemistry, University of California, Riverside, CA 92521, USA

**Keywords:** azide, copper catalysis, multicomponent reactions, tetrasubstituted carbon, triazole

## Abstract

Propargylamines are popular substrates for triazole formation, but tetrasubstituted variants have required multistep syntheses involving stoichiometric amounts of metal. A recent cyclohexanone–amine–silylacetylene coupling forms silyl-protected tetrasubstituted propargylamines in a single copper-catalyzed step. The development of the tandem silyl deprotection–triazole formation reported herein offers rapid access to alpha-tetrasubstituted triazoles. A streamlined two-step approach to this uncommon class of hindered triazoles will accelerate exploration of their therapeutic potential. The superior activity of copper(II) triflate in the formation of triazoles from sensitive alkyne substrates extends to simple terminal alkynes.

## Introduction

1,2,3-Triazoles demonstrate wide spread application in biological systems and drug development [1–12]. Copper-catalyzed azide–alkyne cycloadditions (CuAAC) regioselectively introduce a wide variety of substituents on 1,4-disubstituted 1,2,3-triazoles from the organic azide or terminal alkyne starting materials [1–2]. These Huisgen reactions [13] facilitate rapid drug screening by allowing for tracking in biological systems and the exploration of structure-activity relationships [10,14–19].

**Figure 1:**
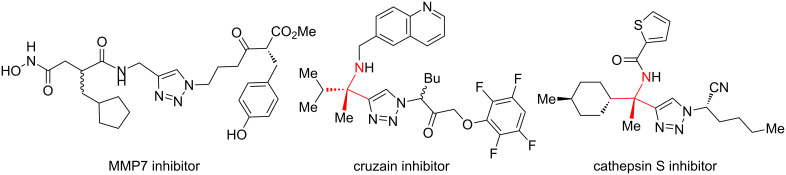
A sampling of propargylamine-derived triazoles with therapeutic effects includes alpha-tetrasubstituted triazoles as cruzain and cathepsin inhibitors.

Propargylamines are a popular choice for the terminal alkyne component and form highly selective inhibitors (Figure 1) [2]. Due to the difficulty of forming tetrasubstituted propargylamines, the incorporation of deprotectable variants into triazoles is extremely rare. The Ellman group demonstrates the power of their chiral sulfinylimine protocol to synthesize propargylamine-derived alpha-tetrasubstituted triazoles (tetrasubstituted carbon bearing amine highlighted in red, Figure 1). One such triazole is a cruzain inhibitor with activity against parasite *Trypanosoma cruzi*, which causes Chagas’ disease [6]. An alpha-tetrasubstituted triazole that inhibits cathepsin S can potentially treat ailments ranging from inflammation to autoimmune disorders [7–8].

The core of the cathepsin S inhibitor is synthesized in six steps. Synthesis and isolation of an *N*-sulfinyl ketimine is followed by stoichiometric alkynylation with a trimethylsilyl-protected alkynyllithium reagent. Removal of the silyl and sulfinyl protecting groups allows for CuAAC with a resin-bound azide. Acylation of the amine followed by dehydration yields the active alpha-tetrasubstituted triazole [7].

The lengthy synthesis of tetrasubstituted propargylamine precursors limits the exploration of such alpha-tetrasubstitued triazoles. The vast majority of three-component couplings produce trisubstituted propargylamines. Copper remains the most popular catalyst for these multicomponent reactions, abbreviated as A^3^ reactions to indicate the Aldehyde, Amine, and Alkyne reaction partners [20–21]. Methods for the corresponding KA^2^, Ketone–Amine–Alkyne, three-component coupling reaction are rare due to the lower electrophilicity and greater steric hindrance of ketones [22–26]. Due to the release of torsional strain when the sp^2^ center in the six-membered ring is attacked, cyclohexanone represents a special case as this cyclic ketone is nearly as reactive as an aldehyde [24].

**Figure 2:**
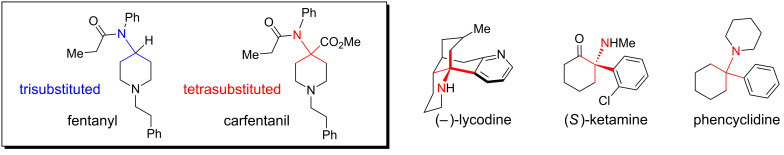
A tetrasubstituted carbon bearing an amine (red) can provide 100-fold increase in activity compared to the trisubstituted carbon bearing an amine (blue).

The resultant tetrasubstituted (red) cyclohexylamine is found in natural alkaloids such as (–)-lycodine (Figure 2), [27] and this motif is also critical to the activity of drugs like ketamine and phencyclidine (1-(1-phenylcyclohexyl)piperidine, PCP) [28]. Tetrasubstituted carbons bearing amines can provide much higher levels of activity than the corresponding trisubstituted center. For example, fentanyl is an anesthetic that is 100 times as powerful as morphine (Figure 2) [29]. By creating a tetrasubstituted variant, the activity is increased two orders of magnitude: carfentanil is over 10,000 times as active as morphine.

## Results and Discussion

### Reaction optimization

**Scheme 1:**
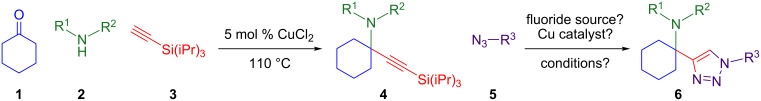
KA^2^ coupling followed by tandem silyl deprotection and triazole formation.

The two-step/three-reaction sequence shown in Scheme 1 would streamline the synthesis of alpha-tetrasubstituted triazoles **6**. In the first step, our solvent-free copper-catalyzed three-component coupling of cyclohexanone (**1**), amines **2**, and alkynes **3** provides high yields of silyl-protected propargylic amines **4** [24–25]. Trimethylsilyl (TMS) acetylene was not stable in the presence of the copper(II) chloride catalyst, and triethylsilylacetylene did not convert cleanly to product. Triisopropylsilyl (TIPS) acetylene was found to be superior to *tert*-butyldimethylsilylacetylene as a source of silylated tetrasubstituted propargylic amines.

Although TMS-protected alkynes have been converted to triazoles via a one-pot silyl deprotection CuAAC reaction [30–33], TIPS-protected alkynes have not. As the triisopropylsilyl protecting group is more difficult to remove than the less hindered trimethylsilyl, conditions for TIPS deprotection include 1.5 equiv of AgF or Cu(OAc)_2_ combined with syringe pump addition of TBAF [34–35]. An additional difficulty is that Ellman’s alpha-tetrasubstituted triazoles are synthesized by CuAAC reaction with desilylated, purified tetrasubstituted propargylic amines [6–8]. Therefore, the goal was to develop the second portion of the sequence in Scheme 1: a tandem deprotection–cycloaddition of tetrasubstituted TIPS-protected propargylamines **4** that would allow them to react in situ with various azides **5** to give hindered triazoles **6**.

**Table 1:** Optimization of silyl deprotection/cycloaddition.

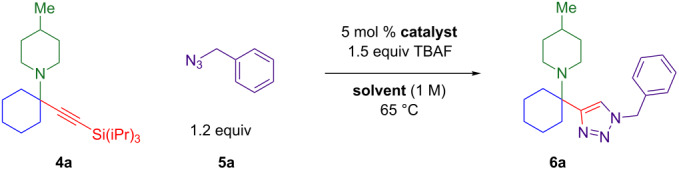				

Entry	Solvent^a^	Catalyst^b^	GC yield (%), 1 h	GC yield (%), 18 h

1	*t*-BuOH/H_2_O (1:1 v/v)	CuCl	21	2
2	DMSO/H_2_O (2:1 v/v)	CuCl	0	0
3	DMF/H_2_O (1:2 v/v)	CuCl	0	0
4	THF/MeOH (1:1 v/v)	CuCl	4	34
5	*t*-BuOH	CuCl	6	62
6	MeOH	CuCl	13	65
7	MeOH	CuBr	62	63
8	MeOH	Cu Powder	24	24
9	MeOH	CuCl_2_	72	90
10	MeOH	CuBr_2_	65	86
11	MeOH	CuF_2_·2H_2_O	69	88
12	MeOH	CuSO_4_·5H_2_O	49	79
13	MeOH	Cu(OAc)_2_·H_2_O	46	82
14	MeOH	Cu(OTf)_2_	39	99

^a^Entries 7–14 were carried out under an atmosphere of argon. ^b^Entries 9–14 with copper(II) sources include 5 mol % of sodium ascorbate reductant.

As a copper(I) catalyst is required for azide–alkyne cycloaddition, the development of a method for the one-pot deprotection/CuAAC began with Cu(I) chloride and a survey of solvents reported [2] for triazole formation (Table 1). TIPS-protected propargylamine **4a** and benzyl azide (**5a**) are heated in the presence of 1.5 equivalents of TBAF (tetrabutylammonium fluoride), 5 mol % CuCl, and the solvent(s) indicated (Table 1, entries 1–6). Aqueous solvent mixtures produce only trace amounts of product at 1 h and 18 h, but methanol and *tert*-butanol provide two-thirds conversion to triazole **6a** after 18 h.

To increase the rate of reaction and to induce complete conversion to **6a**, copper(I) as well as copper(II) sources with an equal amount of sodium ascorbate as the reducing agent were tested in MeOH (Table 1, entries 6–14). All combinations of copper(II) salts with reductant provide higher GC yields (79–99%, Table 1, entries 9–14) than CuCl alone (65%, Table 1, entry 6) at 18 h – corroborating reports that CuAACs are often more efficient with copper(I) catalysts formed from the in situ reduction of copper(II) [2]. After 18 h, copper(II) chloride provides 90% GC yield (Table 1, entry 9), and copper(II) triflate unexpectedly results in quantitative GC yield of **6a** (Table 1, entry 14). Rare in CuAAC reactions, copper(II) triflate is known to form protic acid in solution, which could cause proto-desilylation [36], but this is not observed.

A broader range of green alcohol solvents [37] were assessed in the presence of the most active catalyst, in situ reduced Cu(OTf)_2_. Isopropanol was found to provide faster conversion than methanol, ethanol, and *tert*-butanol at 1 h. A control reaction in isopropanol with in situ reduced copper(II) sources confirms that the triflate is more active than the chloride at 6 h: 67% versus 26% GC yield. Spiking the copper(II) chloride reaction with TfOH provides a similar 30% GC yield. With isopropanol as the solvent, the effect of ambient atmosphere was compared to inert atmosphere. Argon is not necessary, and an atmosphere of nitrogen provides 24% higher GC yield than air. Testing reaction concentration found no significant difference between 0.5 M to 1.0 M isopropanol but increasing the molarity to 2.0 M nearly doubles the GC yield at 18 h from 49% to 87%. Although a lower temperature of 40 °C results in incomplete conversion at 18 h, evidence of product decomposition appears at a higher temperature of 80 °C. A temperature of 60 °C provides two-thirds conversion at 1 h and 85% GC yield of triazole **6a** at 18 h. Finally, when the copper loading is varied from 2.5 mol % to 25 mol % copper(II) triflate with an equivalent loading of sodium ascorbate (NaAsc), GC yields range from 53–63%. However, isolated yields are highest with 10 mol % Cu(OTf)_2_ plus 10 mol % NaAsc as the in situ reductant.

### Substrate scope

With optimized conditions in hand, Table 2 shows the range of triisopropylsilyl cyclohexyl propargylamines that are cleanly converted to the corresponding triazole. Test substrate **6a** is isolated in 66% yield under standard conditions: 10 mol % Cu(OTf)_2_, 10 mol % NaAsc, and 1.5 equiv TBAF in isopropanol (2.0 M) at 65 °C for 6 hours. The *N,N*-diallyl variant **6b** recrystallized in 67% yield is readily deprotectable [38]. This would allow for further elaboration in a similar fashion to the synthesis of cathepsin S inhibitor in Figure 1; *N*-deprotection followed by acylation forms the triazole amide product [6–8]. *N*-[4-(Trifluoromethyl)benzyl]triazole **6c** and morpholinyltriazole **6d** are isolated in 76% and 72% yield, respectively. Cyclopentylamine-derived **6e** and *N*-methylpiperazine-derived **6f** provide additional choices for amine substituents.

**Table 2:** Tetrasubstituted silylpropargylamines form hindered triazole products.

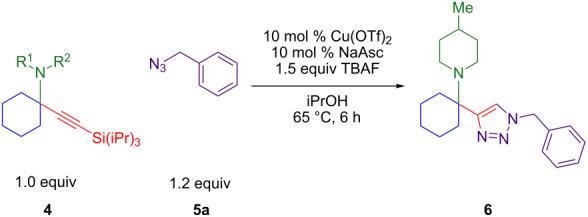	

Propargylic amine **4**	Triazole product **6**

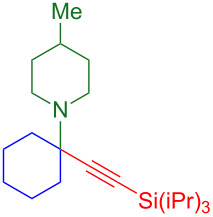 **4a**	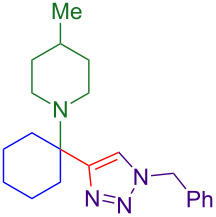 **6a**, 66%
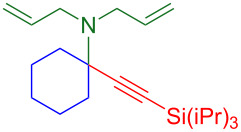	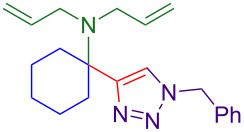 **6b**, 67%
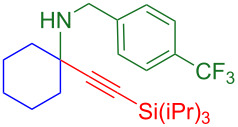	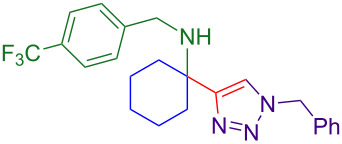 **6c**, 76%
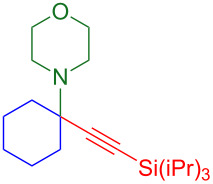	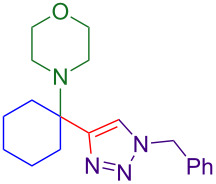 **6d**, 72%
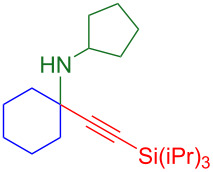	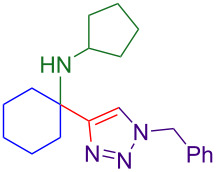 **6e**, 65%
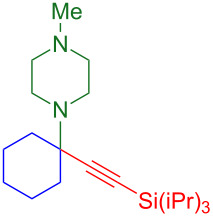	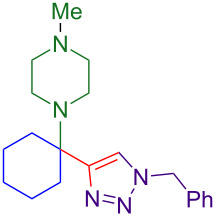 **6f**, 73%

Alternate organic azides were synthesized according to known methods [39–41]. 4-Methylbenzyl azide forms triazole **6g** in a comparable yield to **6a**, but electron-poor 4-(trifluoromethyl)benzyl azide forms triazole **6h** in lower yield. Aryl and alkyl azides display parallel reactivity in the formation of triazoles **6i** and **6j**, and the HCl salt of lipophilic **6j** is isolated cleanly in 56% yield. As TIPS-protected propargylamine **4a** is utilized throughout Table 3, the lower yields observed in some cases are attributed to the intrinsic efficiency of the azide participant.

**Table 3:** Assorted azides for formation of alpha-tetrasubstituted triazoles.

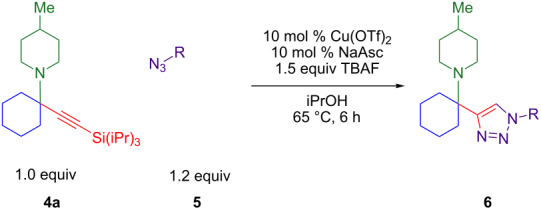	

Azide **5**	Triazole product **6**

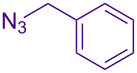 **5a**	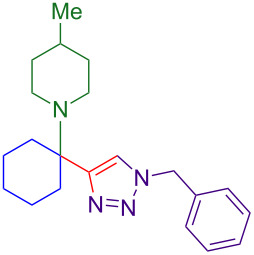 **6a**, 66%
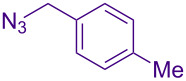	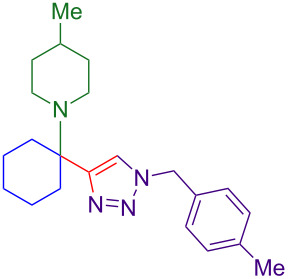 **6g**, 57%
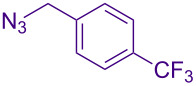	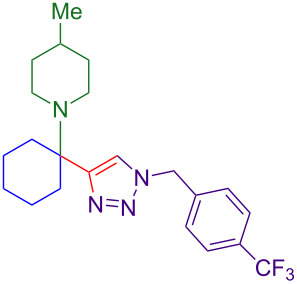 **6h**, 29%
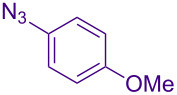	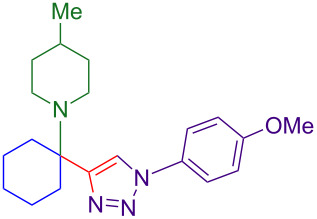 **6i**, 50%
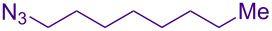	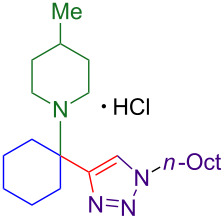 **6j**, 56%

**Scheme 2:**
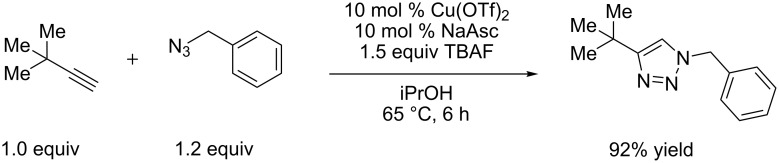
Silyl deprotection/click conditions applied to *tert*-butylacetylene. An identical yield is observed without TBAF.

Control reactions were carried out to assess whether conditions developed for in situ silyl deprotection would otherwise affect the azide when reacting with a simpler terminal alkyne (Scheme 2). As the copper-catalyzed cycloaddition of *tert*-butylacetylene and benzyl azide proceeds in 92% yield, decomposition of the benzyl azide or non-silyl alkyne appears unlikely. Furthermore, this reaction (Scheme 2) proceeds in 92% yield without TBAF. This indicates that the sensitivity of the silyl propargylamine component causes a lower yield of the propargylamine-derived product when reacting with the same benzyl azide under the identical conditions.

**Scheme 3:**
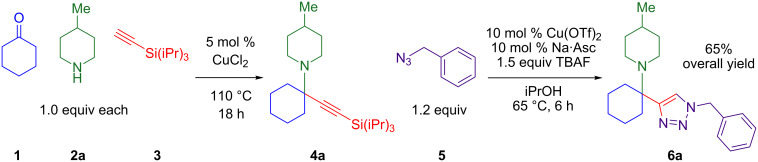
High overall yield of 1,2,3-triazole fully-substituted at the 4-position.

This two-step sequence converts commercially available starting materials into alpha-tetrasubstituted amines via three reactions. It is important to note that triazole **6a** is formed in 65% overall yield (Scheme 3). The first step simply involves heating 5 mol % CuCl_2_ with equimolar amounts of three starting materials, cyclohexanone (**1**), 4-methylpiperidine (**2a**), and TIPS-acetylene (**3**). As there are no added ligands, solvents, promoters, or excess starting materials, the sole byproduct in the formation of tetrasubstituted propargylamine **4a** is one equivalent of water [25]. The second step links silyl-deprotection with azide–alkyne cycloaddition, producing hindered triazoles in 6 hours. This tandem reaction was optimal with a copper(II) triflate and sodium ascorbate (NaAsc) as a mild reductant. The outcome that copper(II) triflate provides the highest yields for the one-pot deprotection/click reaction was unexpected as it is not known as a catalyst for azide–alkyne cycloaddition reactions [1–2].

## Conclusion

A tandem copper-catalyzed silyl deprotection/azide cycloaddition was developed for TIPS-protected tetrasubstituted propargylamines. These substrates are synthesized by a copper-catalyzed ketone–amine–alkyne (KA^2^) coupling that proceeds efficiently with no additives such that an equivalent of water is the sole byproduct. This two-step sequence allows for the incorporation of tetrasubstituted carbons bearing amines at the 4-position of the 1,2,3-triazole core. The overall yield of this three-reaction sequence is high and provides rapid access to hindered triazoles in two steps from commercially available starting materials. Both steps are copper-catalyzed and convert inexpensive starting materials into high-value adducts. As the catalyst combination of copper(II) triflate and sodium ascorbate allows for the inclusion of sensitive and hindered substrates, this unprecedented method should be applicable to the robust and less hindered substrates more common in triazole literature. The power of copper catalysis will accelerate further exploration of the therapeutic potential of alpha-tetrasubstituted triazoles.

## Supporting Information

The Supporting Information features experimental details, compound characterization, and copies of ^1^H and ^13^C NMR spectra of triazoles **6**.

File 1 Experimental details.
