# Supplementary material for: Synthesis of alpha-tetrasubstituted triazoles by copper-catalyzed silyl deprotection/azide cycloaddition
Source: Beilstein J Org Chem. 2015 Aug 14;11:1425–33. doi: 10.3762/bjoc.11.154 (PMC4578341; doi:10.3762/bjoc.11.154)
Supplement: File 1 — Experimental details. [file Beilstein_J_Org_Chem-11-1425-s001.pdf]

Supporting Information
for

Synthesis of alpha-tetrasubstituted triazoles by copper-catalyzed silyl deprotection/azide cycloaddition

Zachary L. Palchak, Paula T. Nguyen, and Catharine H. Larsen*

Address: [1] Department of Chemistry, University of California, Riverside, CA 92521.

Email: Catharine H. Larsen - catharine.larsen@ucr.edu

*Corresponding author

Experimental details

General reagent information

All reactions were set up under ambient atmosphere and carried out in oven-dried screw-cap test-tubes with Teflon seals under an atmosphere of nitrogen. Flash column chromatography was performed using silica gel purchased from Silicycle. $Cu(OTf)_2$ and $CuCl_2$ was purchased from Strem and Acros respectively and used as supplied. Amines were purchased from Acros Organics, Alfa Aesar, or Aldrich and distilled before use. All ketones and alkynes were purchased from Acros Organics, Alfa Aesar or TCI America and were purified by distillation before use. Silyl-protected progargylamines were prepared according to the published literature (Palchak, Z. L.; Lussier, D. J.; Pierce, C. J.; Larsen, C. H. *Green Chem*. **2015**, 17, 1802-1810 DOI: 10.1039/C4GC02318H). Azides were purchased from Alfa Aesar, or Enamine and used as supplied. Additional azides were prepared in accordance with the published literature (Asano, K.; Matsubara, S. *Org. Lett*. **2010**, 12, 4988-4991 DOI: 10.1021/ol101990d).

General analytical information

^{1}H and ^{13}C NMR spectra were measured on a Varian Inova (400 MHz or 500 MHz) spectrometer using $CDCl_3$ as a solvent and trimethylsilane as an internal standard. The following abbreviations are used singularly or in combination to indicate the multiplicity of signals: s - singlet, d - doublet, t - triplet, q - quartet, m - multiplet and br - broad. NMR spectra were acquired at 300 K. Gas chromatography (GC) was carried out on an Agilent Technologies 6850 Network GC System, and dodecane was used as the internal standard. IR spectra were recorded on Perkin Elmer Spectrum One FT-IR Spectrometer. Attenuated total reflection infrared (ATR-IR) was used for analysis with selected absorption maxima reported in wavenumbers (cm^{-1}). Mass spectrometric data was collected on a HP 5989A GC/MS quadrupole instrument. Exact masses were recorded on a Waters GCT Premier TOF instrument using direct injection of samples in acetonitrile into the electrospray source.

General procedure

An oven-dried test tube equipped with a magnetic stir bar was charged with 10 mol% $Cu(OTf)_2$ and 10 mol % sodium ascorbate, capped with a septum, and vacuum purged with nitrogen. Isopropanol was added followed by the silyl-protected propargylamine (1.0 equiv), azide (1.2 equiv), TBAF (1.5 equiv, tetrabutylammonium fluoride, 1 M in THF), and the internal standard dodecane (20 µL). The septum was then replaced under nitrogen pressure with a Teflon-seal screw cap. The reaction was stirred at 65 °C for the indicated time. Upon reaction completion as confirmed by GC analysis, the mixture was cooled to room temperature and the reaction was quenched with ~5 mL H_2O. The reaction was then extracted into diethyl ether, dried over sodium sulfate and concentrated. The resulting oil was placed under high vacuum to remove trace solvent and the resulting product was a brown crude solid. The crude solid was then recrystallized in hexanes to obtain pure triazole product as an off-white solid.

6a: 1-(1-(1-benzyl-1*H*-1,2,3-triazol-4-yl)cyclohexyl)-4-methylpiperidine

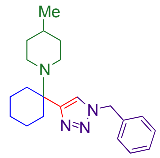

Prepared according to the general procedure: The corresponding silyl-protected propargylamine (360 mg, 1.0 mmol), benzyl azide (158 µL, 1.2 mmol), TBAF in THF (1.5 mL, 1.5 mmol), $Cu(OTf)_2$ (35 mg, 0.1 mmol), and sodium ascorbate (21 mg, 0.1 mmol) afford the title compound as an off white solid in 66% yield (0.224 g, 0.66 mmol) after two recrystallizations from hexanes.
IR (film) 3122, 2913, 1455 cm^{-1}. ^{1}H NMR (400 MHz, $CDCl_3$, 25 °C) δ 7.34 (m, 3H), 7.18 (d, *J* = 7.6 Hz, 2H), 7.15 (s, 1H), 5.51 (s, 2H), 2.99 (d, *J* = 10.8 Hz, 2H), 2.11 (m, 2H), 1.88 (t, *J* = 10 Hz, 2H), 1.67 (t, *J* = 4 Hz, 2H) 1.53 (m, 4H), 1.37 (p, *J* = 5.2 Hz, 2H) 1.15 (m, 5H), 0.81 (d, *J* = 4.8 Hz, 3H) ^{13}C NMR (100 MHz, $CDCl_3$, 25 °C) δ 149.0, 135.4, 129.2, 128.7, 127.8, 121.3, 57.7, 54.0, 45.9, 35.3, 34.5, 31.3, 26.2, 22.4, 22.0. HRMS (ESI) *m*/*z* calcd for $[M+H]^+$ requires 339.2548, found 339.2571.

6b: *N*,*N*-diallyl-1-(1-benzyl-1*H*-1,2,3-triazol-4-yl)cyclohexanamine

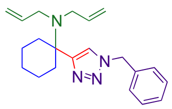

Prepared according to the general procedure: The corresponding silyl-protected propargylamine (360 mg, 1.0 mmol), benzyl azide (158 µL, 1.2 mmol), TBAF in THF (1.5 mL, 1.5 mmol), $Cu(OTf)_2$ (35 mg, 0.1 mmol), and sodium ascorbate (21 mg, 0.1 mmol) afford the title compound as a yellow waxy solid in 67% yield (0.225 g, 0.67 mmol) after chromatography on silica gel (Solvent gradient: 100% hexanes, 10%, 20%, 50% EtOAc in hexanes). IR (film) 3149, 2938, 1456 cm^{-1}.IR (film) 3132, 2932, 1453 cm^{-1}. ^{1}H NMR (400 MHz, $CDCl_3$, 25 °C) δ 7.29 (m, 3H), 7.14 (m, 3H), 5.66 (m, 2H), 5.46 (s, 2H), 4.93 (d, *J* = 17.2 Hz, 2H), 4.82 (d, *J* = 10.4 Hz, 2H), 3.06 (d, *J* = 6.0 Hz, 4H), 2.06 (m, 2H) 1.83 (t, *J*= 10Hz, 2H), 1.62 (m, 2H), 1.32 (m, 2H), 1.17 (m, 2H). ^{13}C NMR (100 MHz, $CDCl_3$, 25 °C) δ 151.4, 139.1, 135.3, 129.2, 128.7, 127.9, 121.4, 114.9, 58.7, 54.1, 51.9, 45.4, 26.1, 22.6. HRMS (ESI) *m*/*z* calcd for $[M+H]^+$ requires 337.2387, found 337.2370.

6c: 1-(1-benzyl-1*H*-1,2,3-triazol-4-yl)-*N*-(4-(trifluoromethyl)benzyl)cyclohexanamine

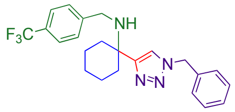

Prepared according to the general procedure: The corresponding silyl-protected propargylamine (437 mg, 1.0 mmol), benzyl azide (158 µL, 1.2 mmol), TBAF in THF (1.5 mL, 1.5 mmol), $Cu(OTf)_2$ (35 mg, 0.1 mmol), and sodium ascorbate (21 mg, 0.1 mmol) afford the title compound as an off white solid in 76% yield (0.316 g, 0.76 mmol) after two recrystallizations from hexanes. IR (film) 3149, 2938, 1456 cm^{-1}. ^{1}H NMR (400 MHz, $CDCl_3$, 25 °C) δ 7.48 (d, *J*= 8 Hz, 2H), 7.35 (d, *J* = 6.4 Hz, 2H), 7.31 (d, *J*= 8 Hz, 2H), 7.21 (s, 1H), 7.18 (d, *J* = 6.4 Hz, 2H), 5.46 (s, 2H), 3.39 (s, 2H), 1.91 (t, *J* = 9.6 Hz, 2H), 1.79 (m, 2H), 1.64 (m, 2H), 1.45 (m, 1H) 1.33 (m, 3H), 1.20 (m, 1H). ^{13}C NMR (100 MHz, $CDCl_3$, 25 °C) δ 154.6, 145.6, 135.1, 129.3(2 overlapped peaks), 128.8, 128.5, 128.0, 125.3(2 overlapped peaks), 120.8, 54.7, 54.2, 46.5, 36.3, 26.0, 22.1. HRMS (ESI) *m*/*z* calcd for $[M+H]^+$ requires 415.2104, found 415.2115.

6d: 4-(1-(1-benzyl-1*H*-1,2,3-triazol-4-yl)cyclohexyl)morpholine

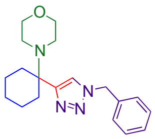

Prepared according to the general procedure: The corresponding silyl-protected propargylamine (349 mg, 1.0 mmol), benzyl azide (158 µL, 1.2 mmol), TBAF in THF (1.5 mL, 1.5 mmol), $Cu(OTf)_2$ (35 mg, 0.1 mmol), and sodium ascorbate (21 mg, 0.1 mmol) afford the title compound as a white solid in 72% yield (0.234 g, 0.72 mmol) after two recrystallizations from hexanes. IR (film) 3125, 2928, 1449 cm^{-1}. ^{1}H NMR (400 MHz, $CDCl_3$, 25 °C) δ 7.34 (m, 3H), 7.20 (dd, *J*= 7.6 Hz, 2 Hz, 2H), 7.16 (s, 1H), 5.51 (s, 2H), 3.62 (t, *J* = 4.4 Hz, 4H), 2.34 (m, 4H), 2.04 (m, 2H), 1.90 (t, *J* = 10 Hz, 2H) 1.68 (m, 2H), 1.40 (t, *J* = 5.6 Hz, 2H) 1.23 (m, 2H). ^{13}C NMR (100 MHz, $CDCl_3$, 25 °C) δ 148.5, 135.1, 129.3, 128.8, 127.9, 121.3, 67.8, 57.4, 54.1, 46.1, 33.7, 26.1, 22.1. HRMS (ESI) *m*/*z* calcd for $[M+H]^+$ requires 327.2179, found 327.2187.

6e: 1-(1-benzyl-1*H*-1,2,3-triazol-4-yl)-*N*-cyclopentylcyclohexanamine

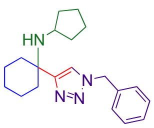

Prepared according to the general procedure: The corresponding silyl-protected propargylamine (348 mg, 1.0 mmol), benzyl azide (158 µL, 1.2 mmol), TBAF in THF (1.5 mL, 1.5 mmol), $Cu(OTf)_2$ (35 mg, 0.1 mmol), and sodium ascorbate (21 mg, 0.1 mmol) afford the title compound as an off white solid in 65% yield (0.316 g, 0.76 mmol) after chromatography on silica gel (Solvent gradient: 100% hexanes, 10%, 20%, 50% EtOAc in hexanes) and finally recrystallized out of hexanes. IR (film) 3149, 2938, 1456 cm^{-1}.IR (film) 3124, 2940, 1445 cm^{-1}. ^{1}H NMR (400 MHz, $CDCl_3$, 25 °C) δ 7.34 (m, 3H), 7.25 (s, 1H), 7.20 (dd, *J*= 7.6 Hz, 2 Hz, 2H), 5.51 (s, 2H), 2.72 (p, *J* = 8.4 Hz, 2H), 2.07 (m, 2H), 1.67 (m, 4H), 1.51 (m, 4H) 1.38 (m, 2H), 1.30 (m, 4H) 1.05 (m, 2H). ^{13}C NMR (100 MHz, $CDCl_3$, 25 °C) δ 154.8, 135.3, 129.2, 128.7, 127.9, 121.2, 54.9, 54.5, 54.1, 37.1, 35.4, 26.1, 23.9, 22.6. HRMS (ESI) *m*/*z* calcd for $[M+H]^+$ requires 325.2387, found 325.2400.

6f: 1-(1-(1-benzyl-1*H*-1,2,3-triazol-4-yl)cyclohexyl)-4-methylpiperazine

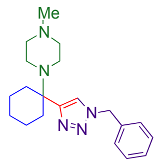

Prepared according to the general procedure: The corresponding silyl-protected propargylamine (362 mg, 1.0 mmol), benzyl azide (158 µL, 1.2 mmol), TBAF in THF (1.5 mL, 1.5 mmol), $Cu(OTf)_2$ (35 mg, 0.1 mmol), and sodium ascorbate (21 mg, 0.1 mmol) afford the title compound as an off white solid in 73% yield (0.250 g, 0.73 mmol) after two recrystallizations from hexanes.IR (film) 3124, 2929, 1452 cm^{-1}. ^1H NMR (500 MHz, $CDCl_3$, 25 °C) δ 7.37 (m, 3H), 7.24 (d, *J* = 7.0 Hz, 2H), 7.18 (s, 1H), 5.50 (s, 2H), 2.4 (m, 4H), 2.21 (s, 3H), 2.11 (m, 2H), 1.93 (t, *J* = 10 Hz, 2H) 1.70 (m, 2H), 1.42 (p, *J* = 5.5 Hz, 2H) 1.25 (m, 2H). ^{13}C NMR (125 MHz, $CDCl_3$, 25 °C) δ 148.1, 134.8, 129.0, 128.5, 127.9, 121.0, 57.1, 55.8, 53.9, 45.9, 45.1, 33.9, 26.0, 22.0. HRMS (ESI) *m*/*z* calcd for $[M+H]^+$ requires 340.2496, found 340.2493.

6g: 4-methyl-1-(1-(1-(4-methylbenzyl)-1*H*-1,2,3-triazol-4-yl)cyclohexyl)piperidine

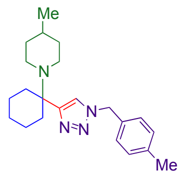

Prepared according to the general procedure: The corresponding silyl-protected propargylamine (360 mg, 1.0 mmol), 4-methyl benzyl azide (176 mg, 1.2 mmol), TBAF in THF (1.5 mL, 1.5 mmol), $Cu(OTf)_2$ (35 mg, 0.1 mmol), and sodium ascorbate (21 mg, 0.1 mmol) afford the title compound as an off white solid in 57% yield (0.201 g, 0.57 mmol) after two recrystallizations from hexanes. IR (film) 3119, 2915, 1458 cm^{-1}. ^1H NMR (400 MHz, $CDCl_3$, 25 °C) δ 7.15 (d, *J*= 8 Hz, 2H), 7.13 (s, 1H), 7.09 (d, *J*= 8 Hz, 2H), 5.47 (s, 2H), 2.99 (d, *J* = 10.8 Hz, 2H), 2.33 (s, 3H), 2.11 (m, 2H), 1.88 (t, *J* = 12.4 Hz, 2H) 1.69 (m, 2H), 1.54 (m, 2H), 1.37 (p, *J* = 5.6 Hz, 2H), 1.20 (m, 2H), 1.19 (m, 3H), 0.81 (d, *J* = 5.2 Hz, 3H). ^{13}C NMR (100 MHz, $CDCl_3$, 25 °C) δ 148.9, 138.5, 132.3, 129.9, 127.9, 121.2, 57.7, 53.9, 46.0, 35.4, 34.5, 31.2, 26.2, 22.4, 22.0, 21.3. HRMS (ESI) *m*/*z* calcd for $[M+H]^+$ requires 353.2700, found 353.2689.

6h: 4-methyl-1-(1-(1-(4-(trifluoromethyl)benzyl)-1*H*-1,2,3-triazol-4-yl)cyclohexyl)piperidine

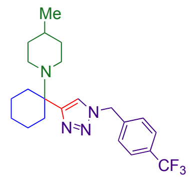

Prepared according to the general procedure: The corresponding silyl-protected propargylamine (360 mg, 1.0 mmol), 4-trifluoromethyl benzyl azide (210 mg, 1.2 mmol), TBAF in THF (1.5 mL, 1.5 mmol), $Cu(OTf)_2$ (35 mg, 0.1 mmol), and sodium ascorbate (21 mg, 0.1 mmol) afford the title compound as an off white solid in 29% yield (0.117 g, 0.29 mmol) after two recrystallizations from hexanes. IR (film) 3118, 2939, 1456 cm^{-1}. ^1H NMR (500 MHz, $CDCl_3$, 25 °C) δ 7.64 (d, *J*= 8 Hz, 2H), 7.32 (d, *J* = 8 Hz, 2H), 7.24 (s, 1H), 5.61 (s, 2H), 3.04 (d, *J* = 10 Hz, 2H), 2.14 (m, 2H), 1.95 (t, *J* = 11 Hz, 2H), 1.72 (m, 2H) 1.58 (t, *J*= 9.5 Hz, 4H), 1.42 (t, *J*= 6 Hz, 2H) 1.24 (m, 2H), 1.15 (m, 2H), 0.85 (d, *J*= 4.5 Hz, 3H). ^{13}C NMR (125 MHz, $CDCl_3$, 25 °C) δ 149.2, 139.1, 130.6, 127.7, 126.0, 121.2, 57.5, 53.2, 45.8, 35.1, 34.2, 31.1, 26.0, 22.1, 21.8. HRMS (ESI) *m*/*z* calcd for $[M+H]^+$ requires 407.2417, found 407.2391.

6i: 1-(1-(1-(4-methoxyphenyl)-1*H*-1,2,3-triazol-4-yl)cyclohexyl)-4-methylpiperidine

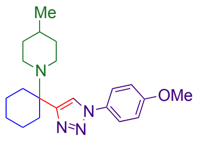

Prepared according to the general procedure: The corresponding silyl-protected propargylamine (360 mg, 1.0 mmol), 1-azido-4-methoxy benzene (178 µL, 1.2 mmol), TBAF in THF (1.5 mL, 1.5 mmol), $Cu(OTf)_2$ (35 mg, 0.1 mmol), and sodium ascorbate (21 mg, 0.1 mmol) afford the title compound as an off white beige solid in 50% yield (0.177 g, 0.50 mmol) after two recrystallizations from hexanes. IR (film) 3132, 2922, 1513 cm^{-1}. ^1H NMR (400 MHz, $CDCl_3$, 25 °C) δ 7.64 (d, *J*= 78.8 Hz, 2H), 7.62 (s, 1H), 6.99 (d, *J*= 9.2 Hz, 2H), 3.85 (s, 3H), 3.08 (d, *J* = 10.4 Hz, 2H), 2.24 (m, 2H), 1.96 (t, *J* = 10.8 Hz, 2H), 1.70 (m, 4H) 1.57 (m, 2H), 1.43 (m, 2H) 1.28 (m, 3H), 1.16 (m, 2H), 0.82 (d, *J* = 5.6 Hz, 3H). ^{13}C NMR (100 MHz, $CDCl_3$, 25 °C) δ 159.7, 149.0, 131.0, 122.1, 119.5, 114.8, 57.7, 55.8, 46.0, 35.4, 34.5, 31.2, 26.2, 22.4, 22.1. HRMS (ESI) *m/z* calcd for $[M+H]^+$ requires 355.2492, found 355.2505.

6j: 4-methyl-1-(1-(1-octyl-1*H*-1,2,3-triazol-4-yl)cyclohexyl)piperidin-1-ium chloride

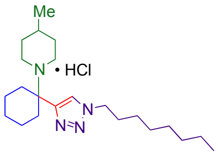

Prepared according to the general procedure: The corresponding silyl-protected propargylamine (360 mg, 1.0 mmol), 1-azido-octane (186 mg, 1.2 mmol), TBAF in THF (1.5 mL, 1.5 mmol), $Cu(OTf)_2$ (35 mg, 0.1 mmol), and sodium ascorbate (21 mg, 0.1 mmol) afford the title compound as an off white solid in 56% yield (0.201 g, 0.56 mmol) after chromatography on silica gel (solvent gradient: 100% hexanes, 10%, 20%, 50%, 100% EtOAc in hexanes) and finally precipitated out of diethyl ether as the HCl salt. IR (film) 3152, 2925, 2465, 1450 cm-1. 1H NMR (500 MHz, CDCl3, 25 °C) δ 11.48 (s, 1H), 8.01 (s, 1H), 4.41 (t, J= 7 Hz, 2H), 3.60 (d, J= 11 Hz, 2H), 2.84 (d, J = 12 Hz, 2H), 2.50 (d, J = 10.5 Hz, 2H), 2.34 (t, J = 11.5 Hz, 2H), 2.13 (q, J = 12.5 Hz, 2H), 1.95 (m, 2H), 1.83 (d, J = 13 Hz, 2H), 1.72 (d, J = 14 Hz, 2H), 1.57 (d, J = 11 Hz, 1H), 1.29 (m, 14H), 1.17 (d, J = 11 Hz, 1H), 0.96 (d, J = 6 Hz, 3H) 0.87 (t, J= 6.5 Hz, 3H). 13C NMR (125 MHz, CDCl3, 25 °C) δ 142.2, 125.6, 67.0, 50.8, 47.2, 31.6, 30.9, 30.5, 30.1, 29.8, 29.0, 28.8, 26.5, 24.4, 22.9, 22.5, 20.8, 14.0. HRMS (ESI) m/z calcd for [M+H]+ requires 361.3326, found 361.3316.

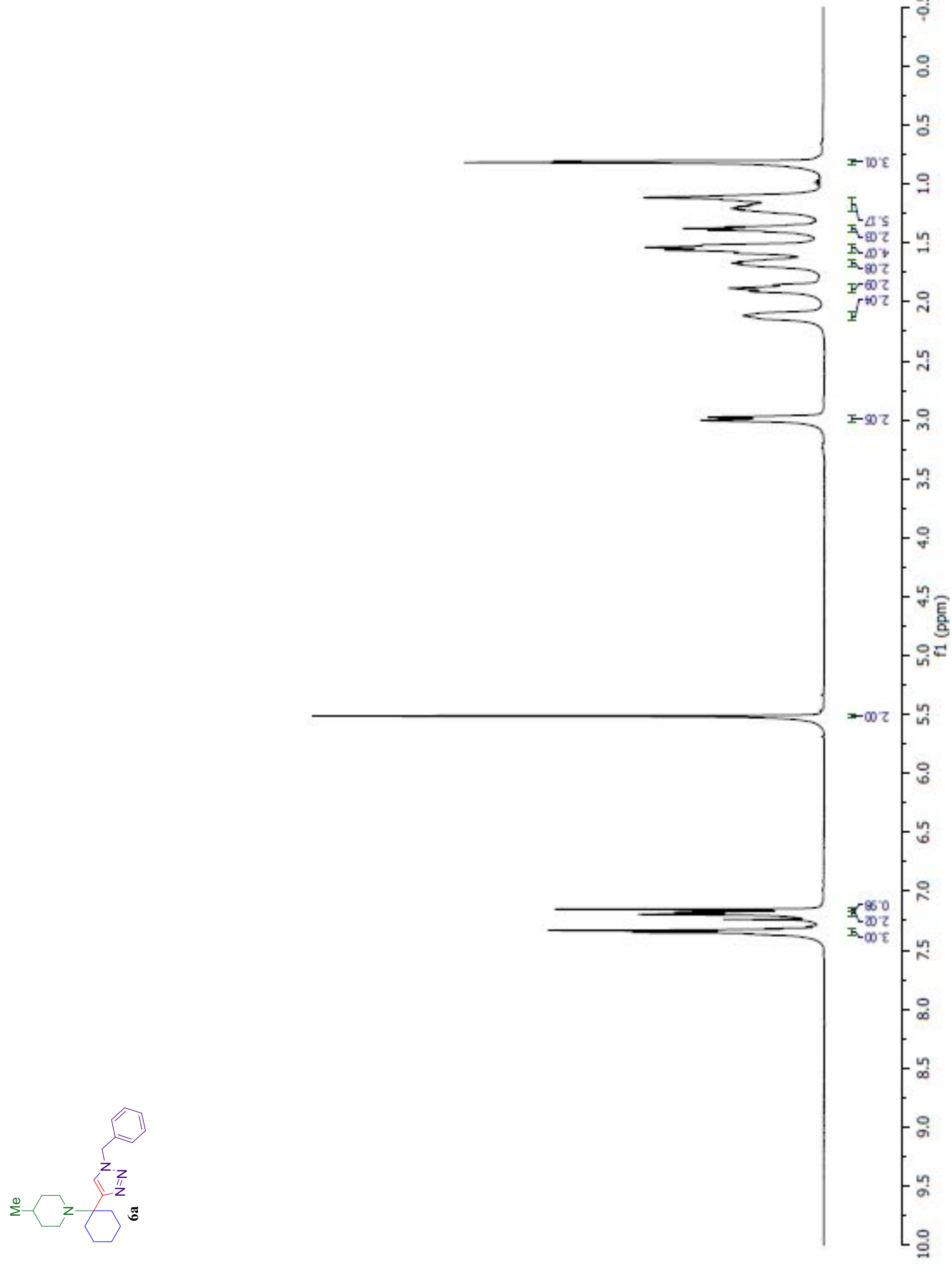

f1 (ppm)
6a
Me

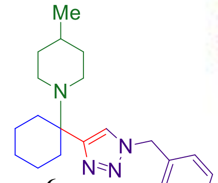

6a

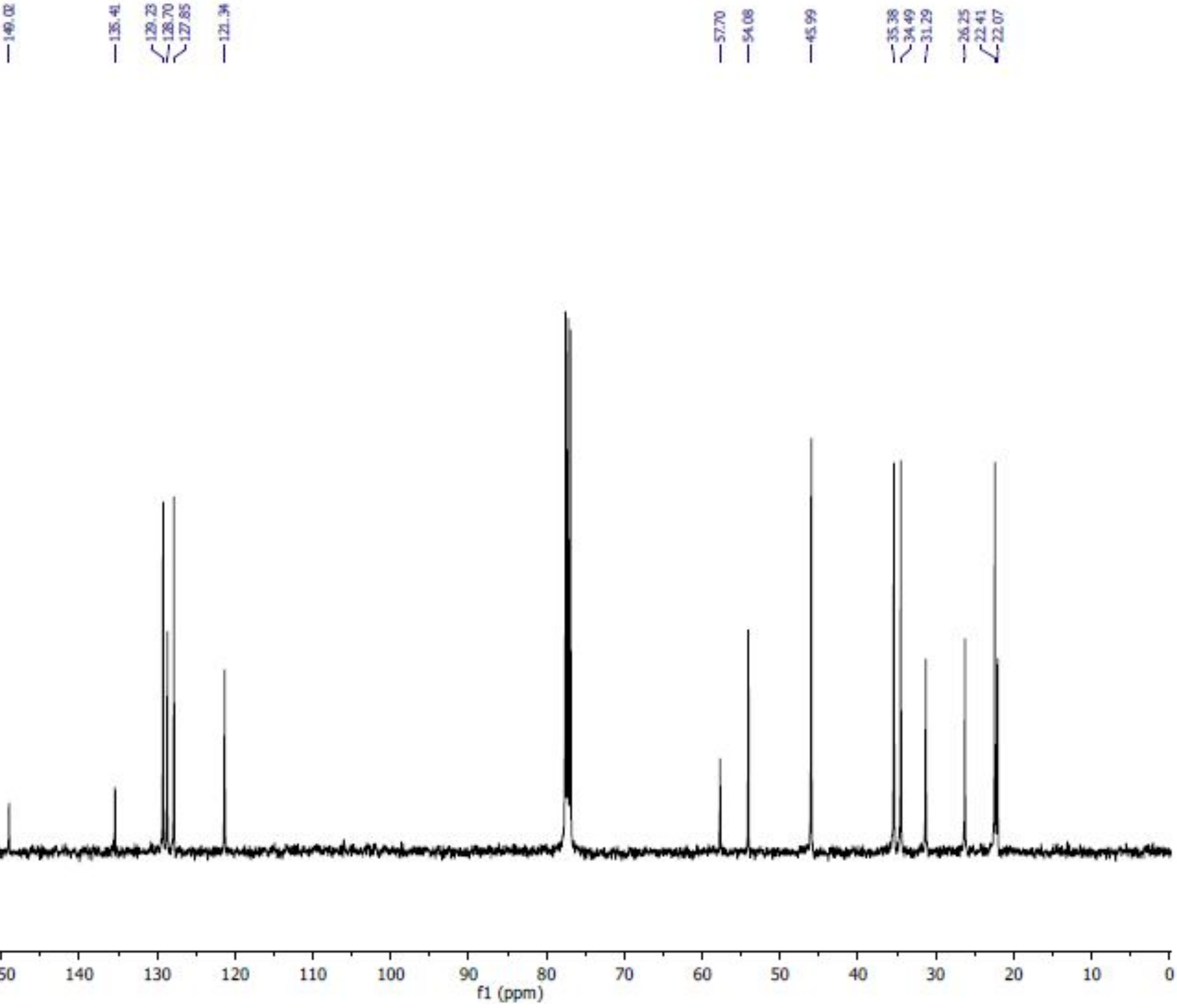
149.02
135.41
129.23
128.70
127.85
121.34
57.70
54.08
45.99
35.38
34.49
31.29
26.25
22.41
22.07
160
150
140
130
120
110
100
90
80
70
60
50
40
30
20
10
0
f1 (ppm)

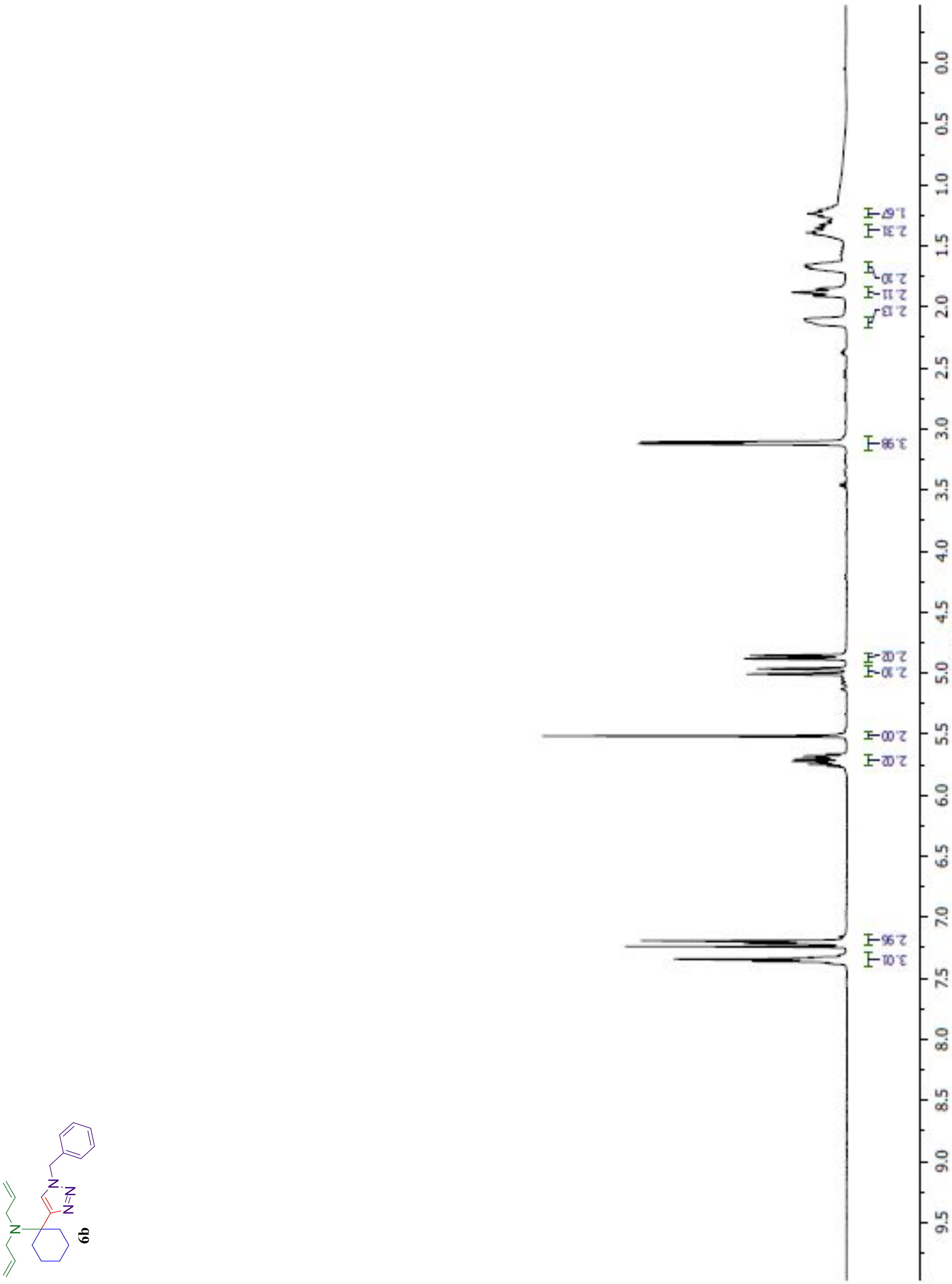

6b

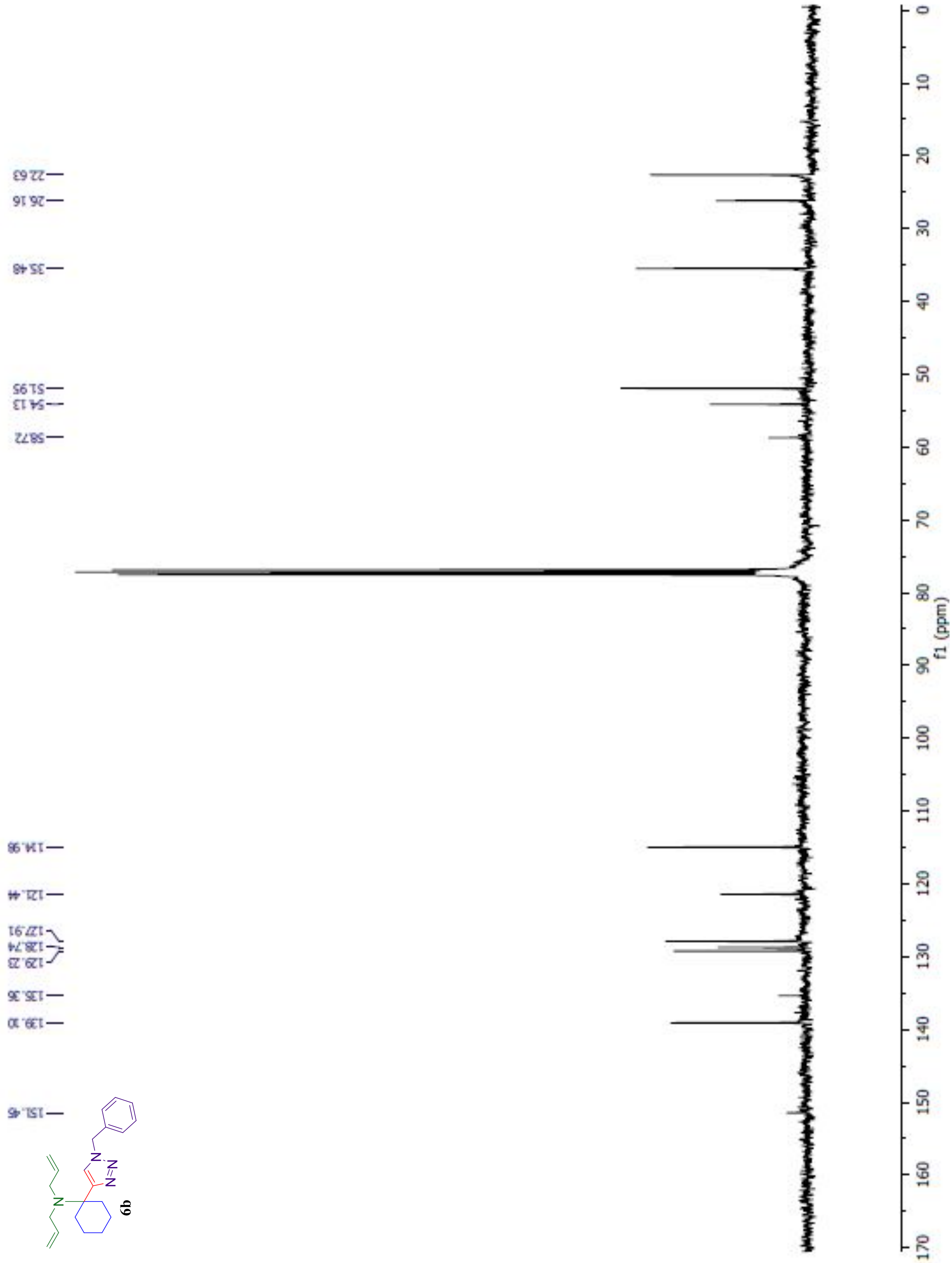

22.63
26.16
35.48
51.95
54.13
58.72
114.98
121.44
127.91
128.74
129.23
135.36
139.10
151.45
f1 (ppm)

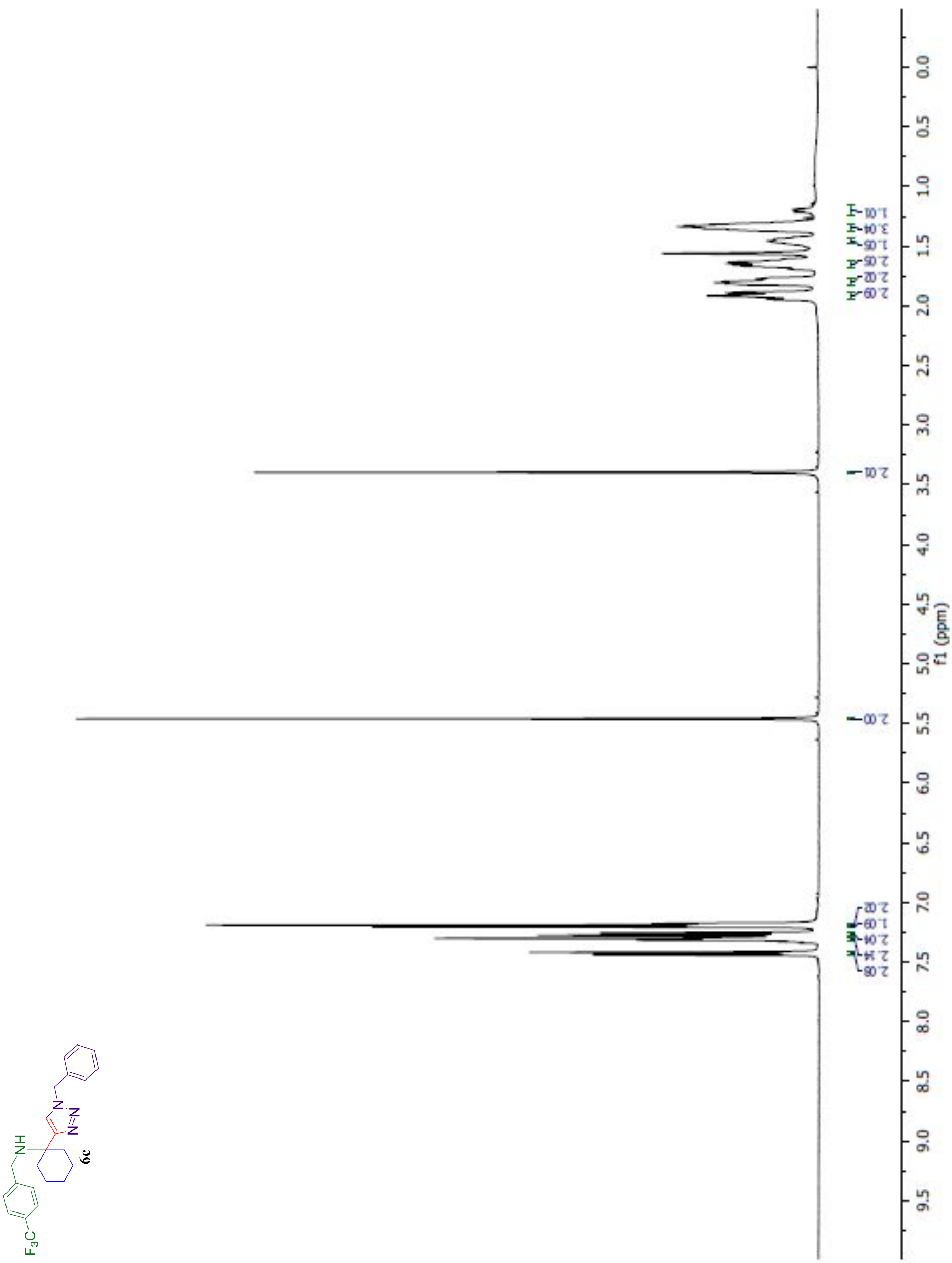
6c
f1 (ppm)

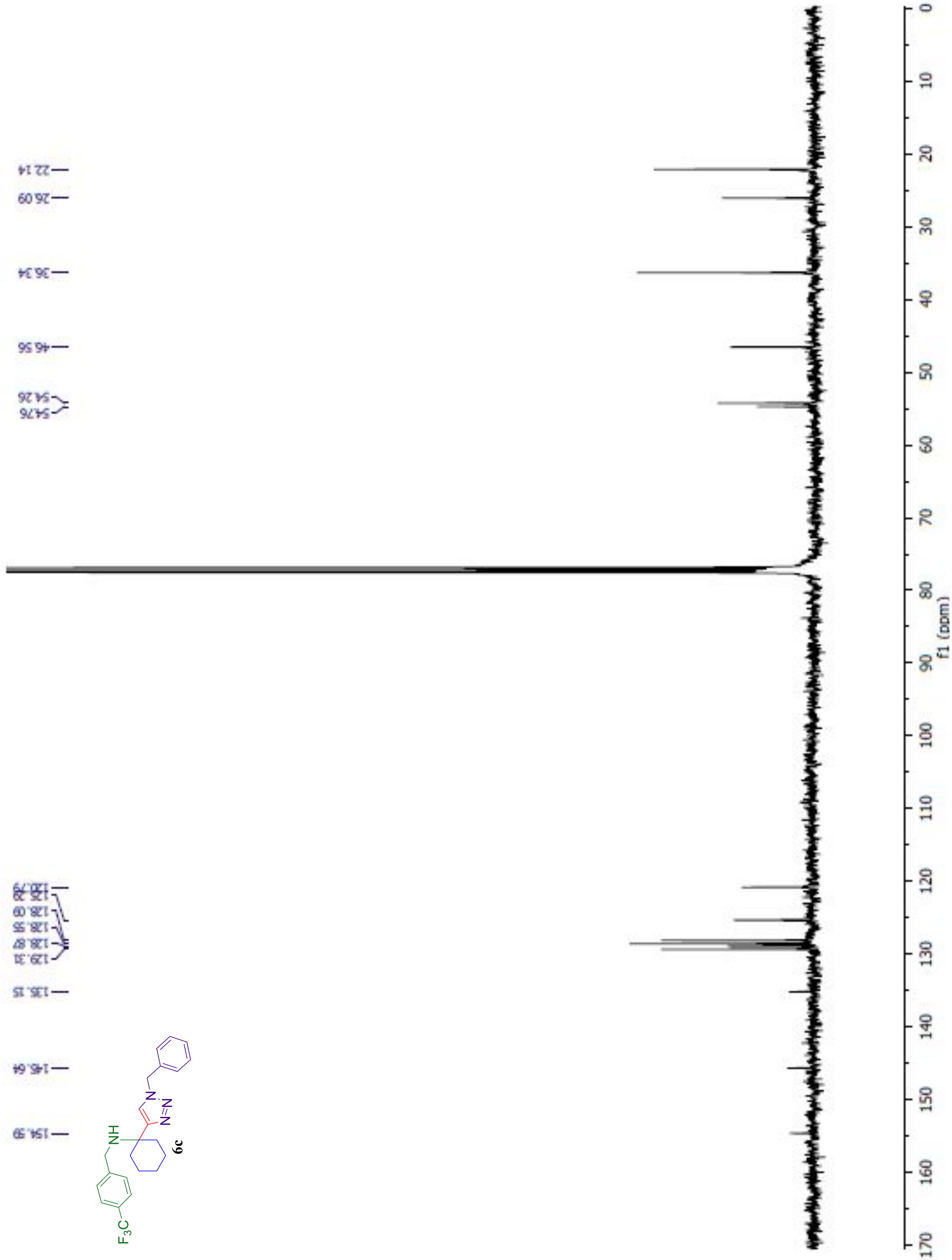
22.14
26.09
36.34
46.56
54.26
54.76
128.09
128.55
128.87
129.31
135.15
145.64
154.59
NH
F3C
6c
f1 (ppm)

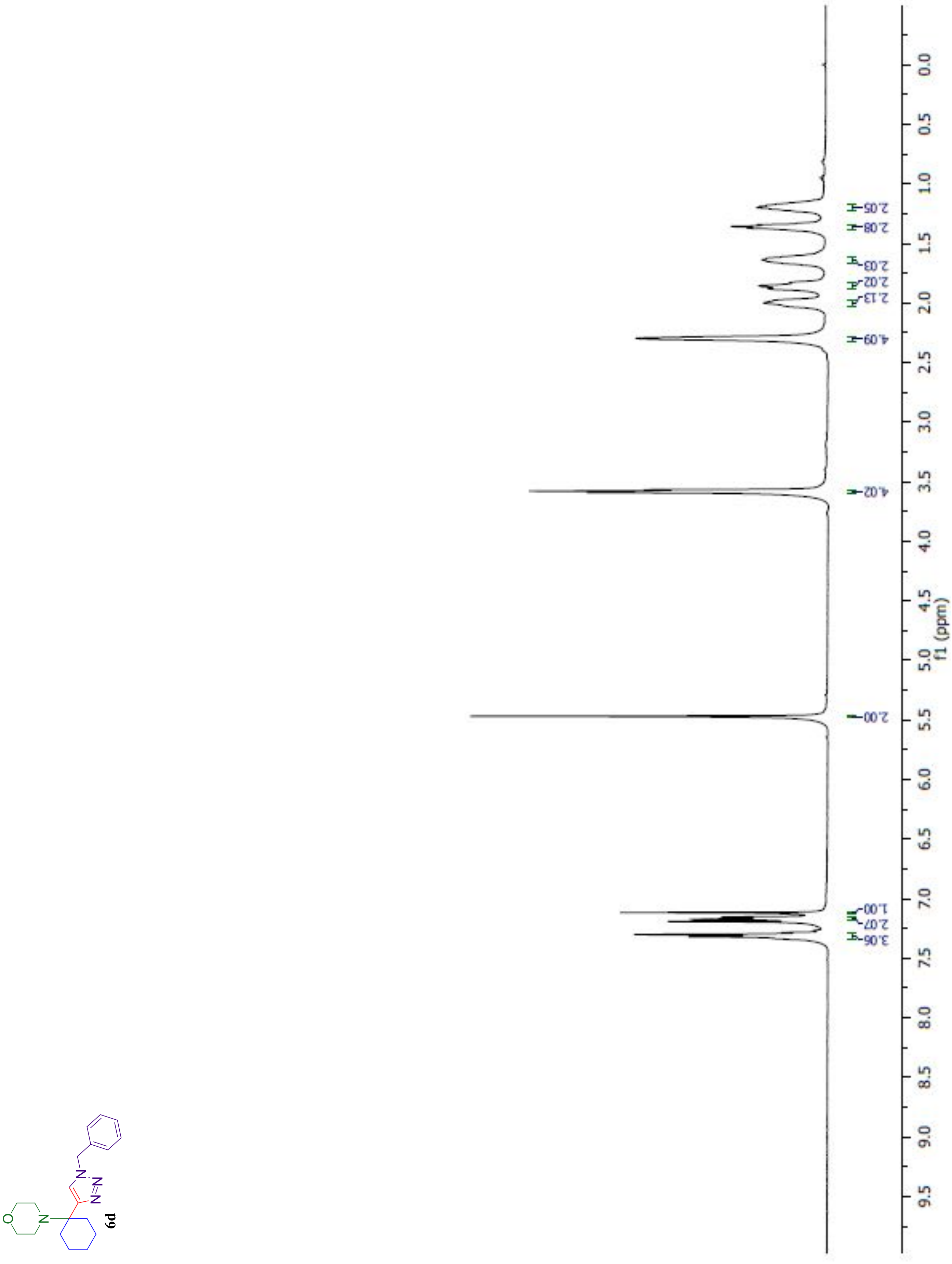
6d
O
N
N
N=N
f1 (ppm)
0.0 0.5 1.0 1.5 2.0 2.5 3.0 3.5 4.0 4.5 5.0 5.5 6.0 6.5 7.0 7.5 8.0 8.5 9.0 9.5
2.05
2.08
2.03
2.02
2.13
4.09
4.02
2.00
1.00
2.07
3.06

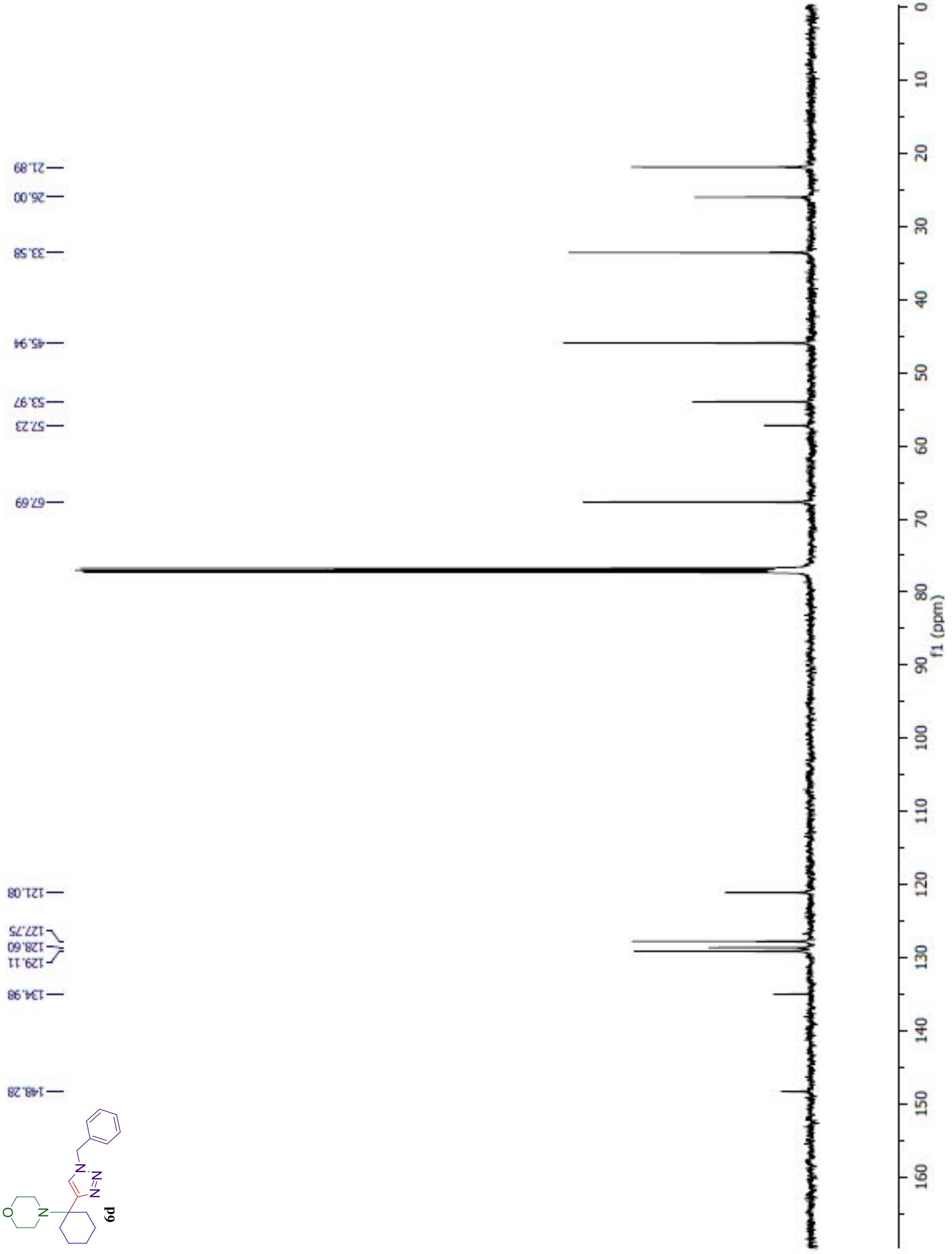

21.89
26.00
33.58
45.94
53.97
57.23
67.69
121.08
127.75
128.60
129.11
134.98
148.28
9d
f1 (ppm)

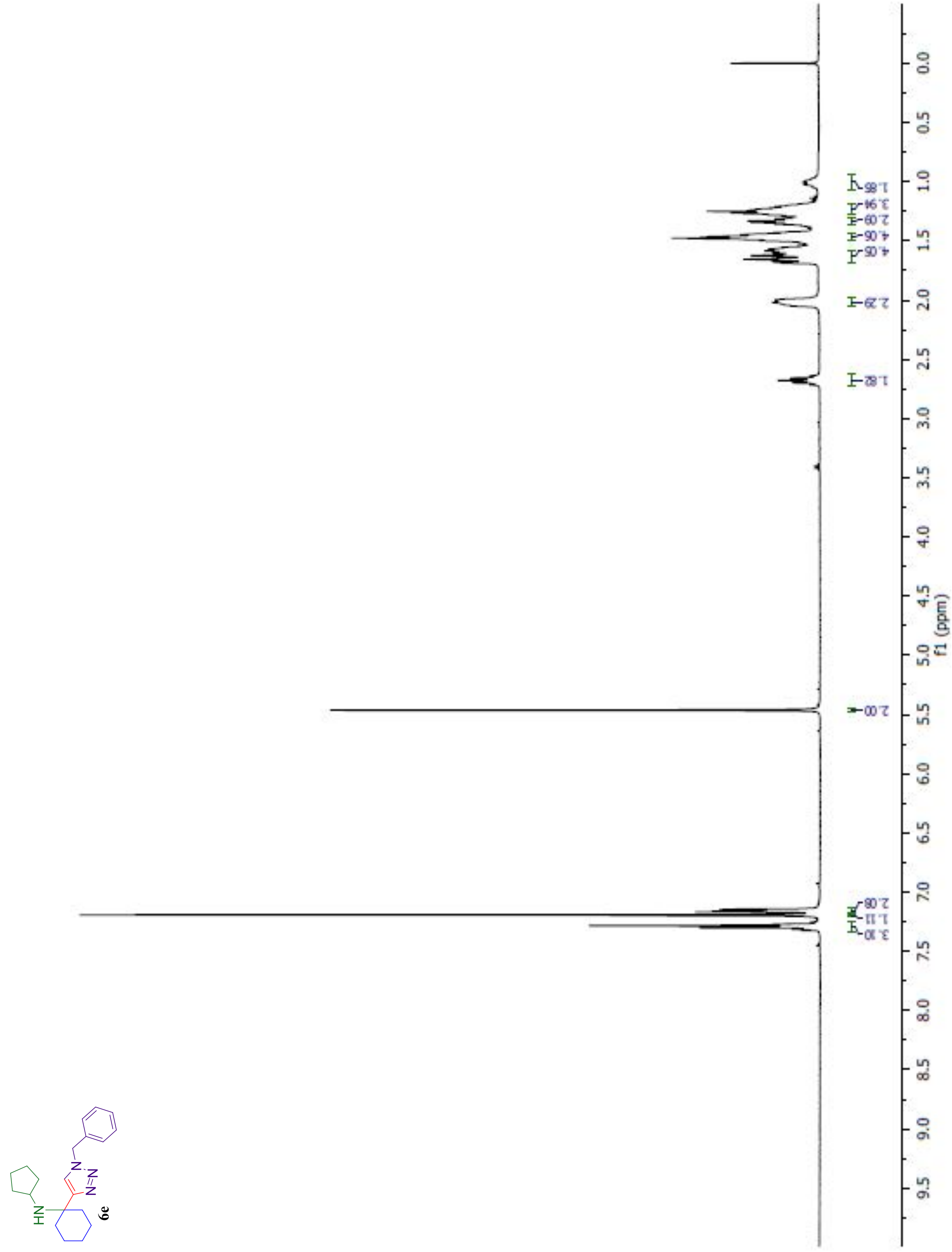

6e

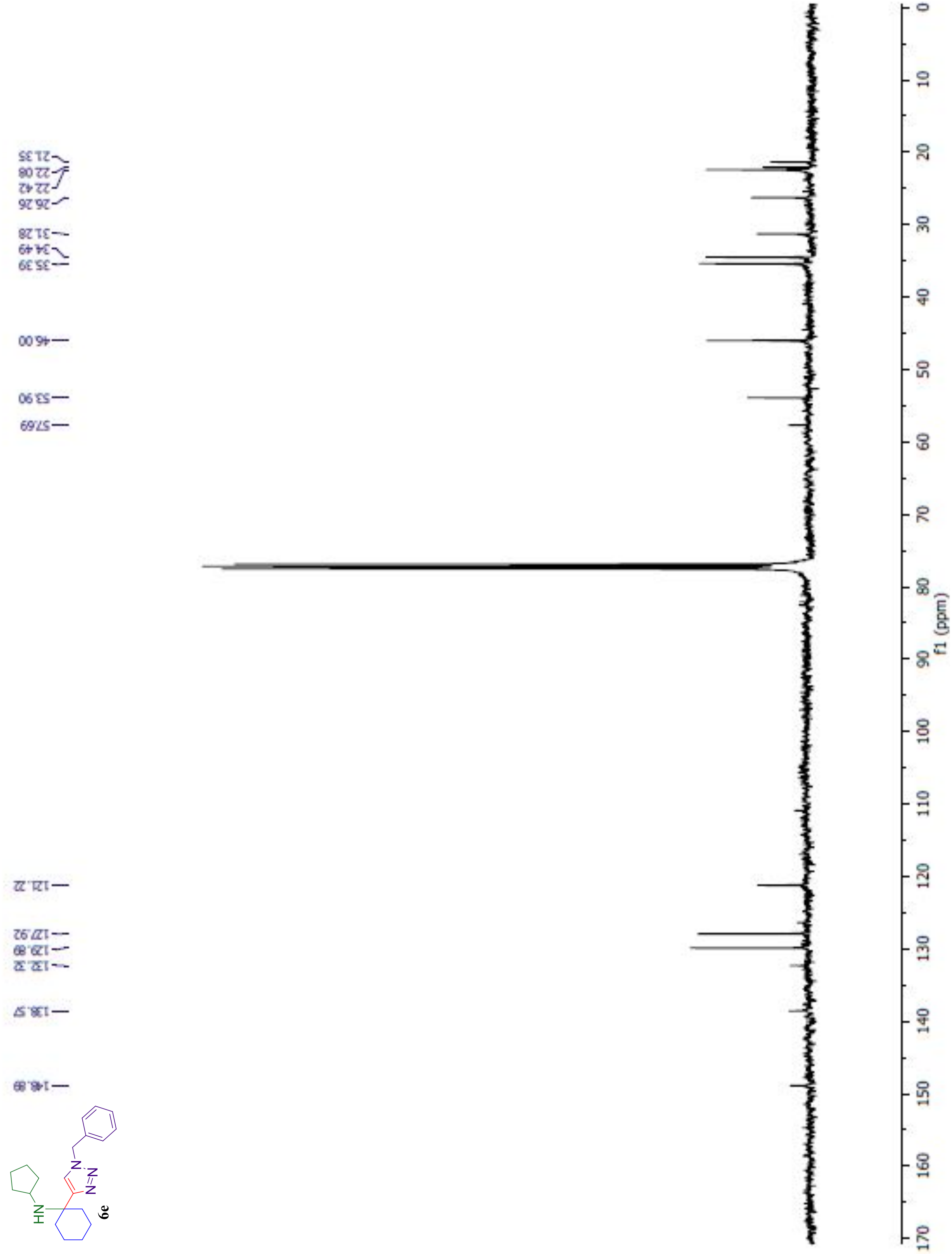

21.35
22.08
22.42
26.26
31.28
34.49
35.39
46.00
53.90
57.69
121.22
127.92
129.89
132.32
138.57
148.89
HN
N
N=N
6e
0
10
20
30
40
50
60
70
80
90
100
110
120
130
140
150
160
170
f1 (ppm)

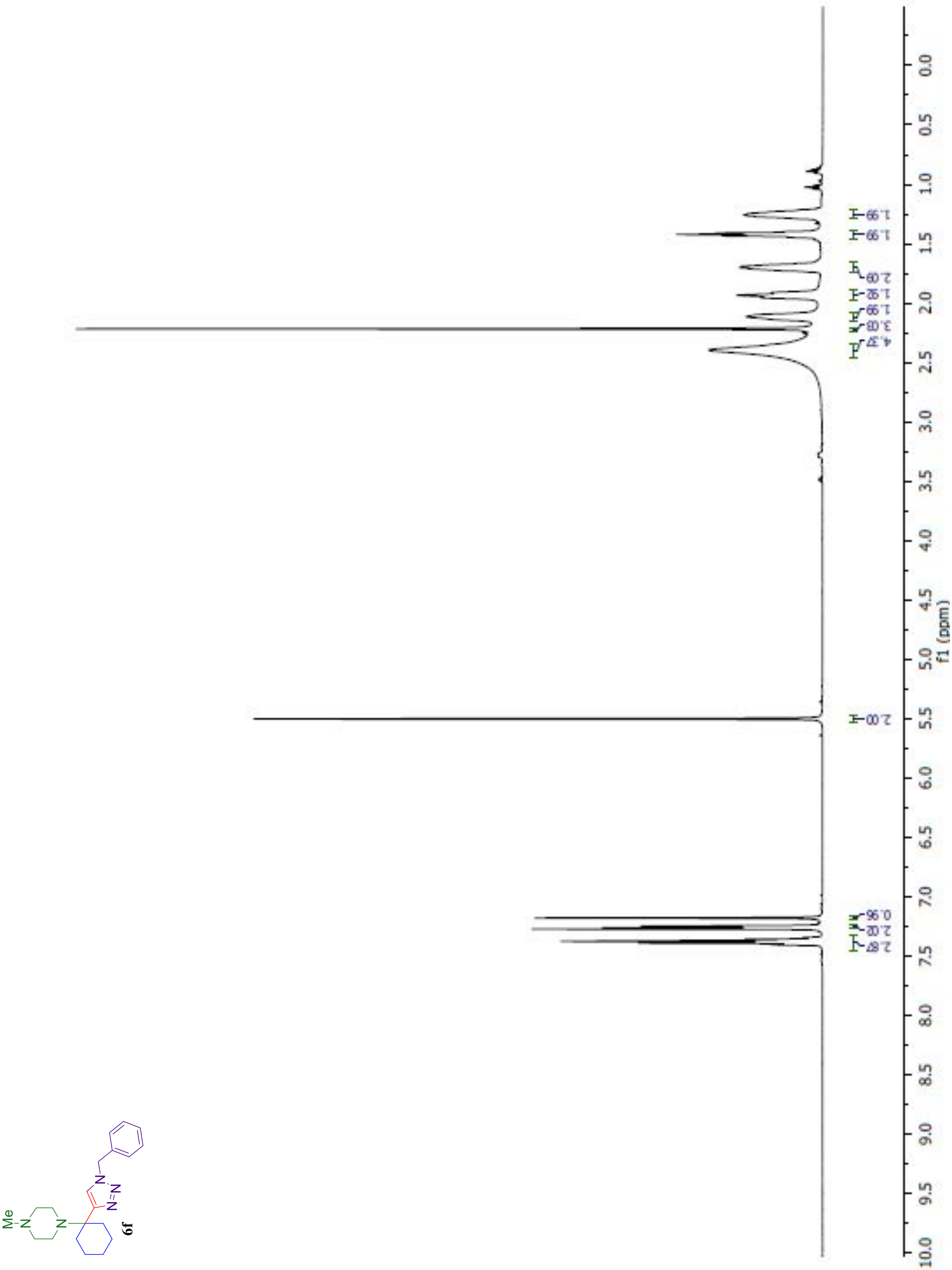
f1 (ppm)
6f

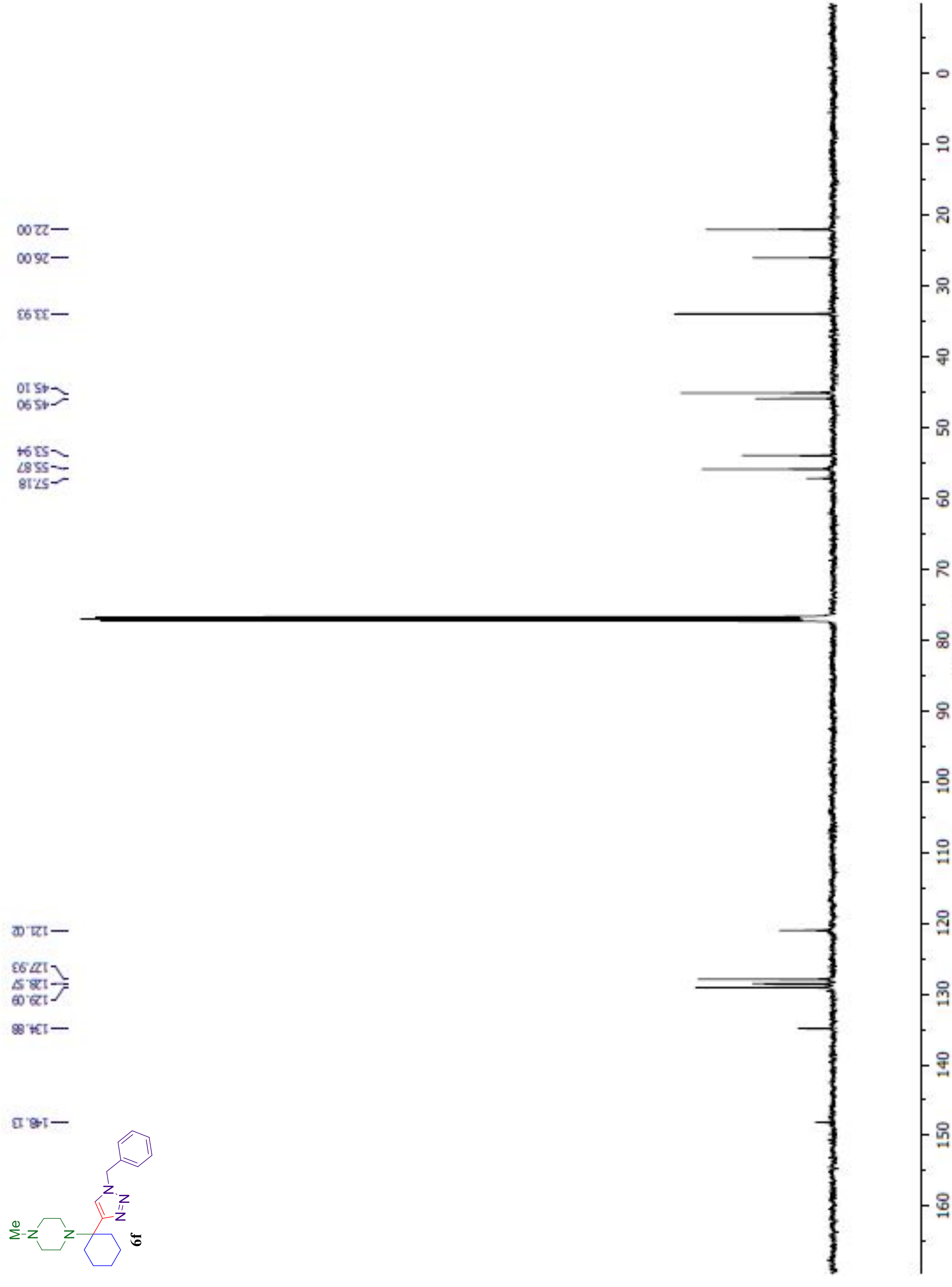
22.00
26.00
33.93
45.10
45.90
53.94
55.87
57.18
121.02
127.93
128.57
129.09
134.88
148.13
Me
N
N
N
N=N
6f
f1 (ppm)

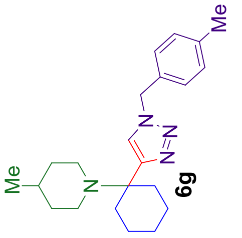
Me
N
N
N=N
Me

6g

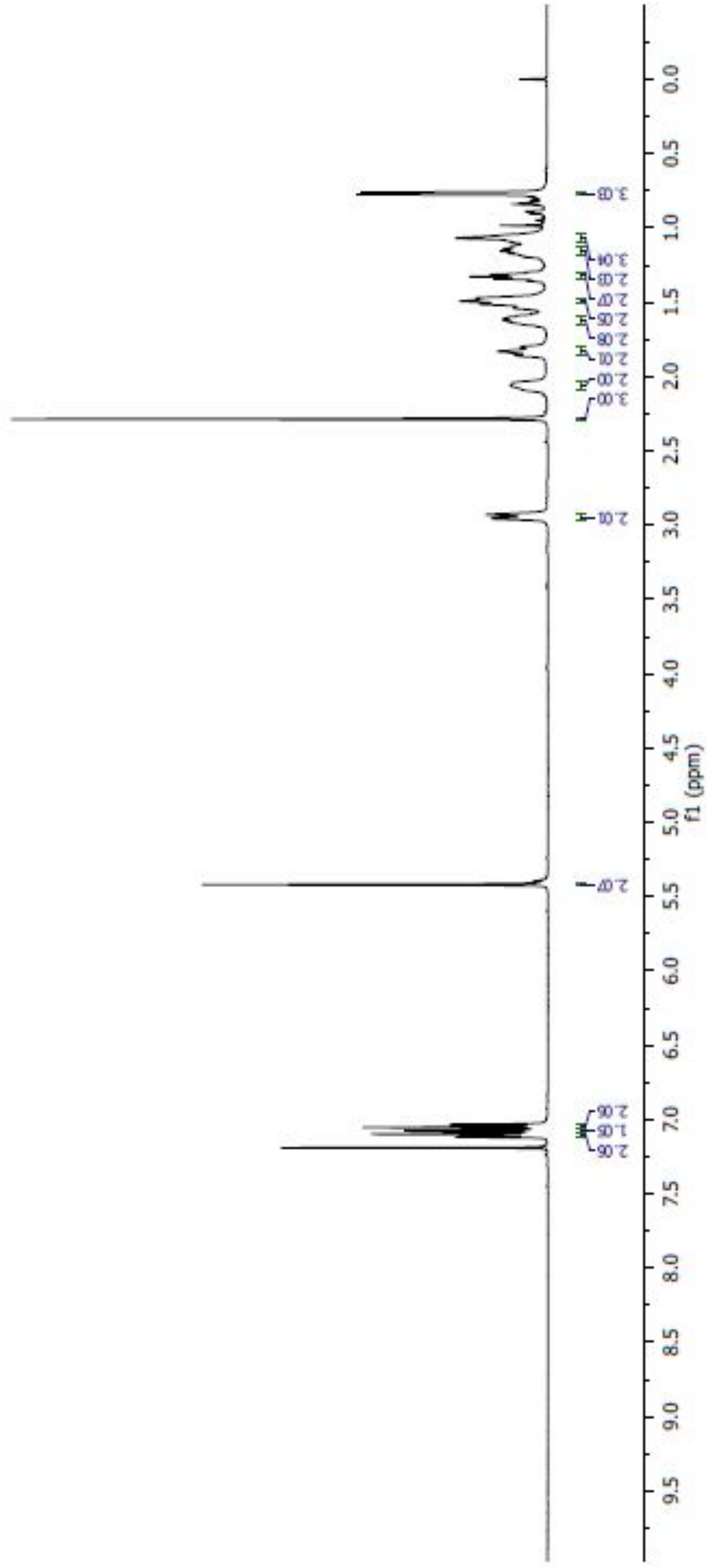
0.0
0.5
1.0
1.5
2.0
2.5
3.0
3.5
4.0
4.5
5.0
5.5
6.0
6.5
7.0
7.5
8.0
8.5
9.0
9.5
f1 (ppm)

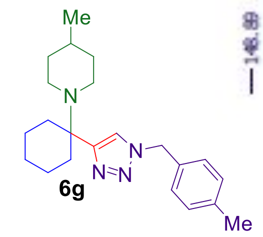
Me
N
N
N
N
Me

6g

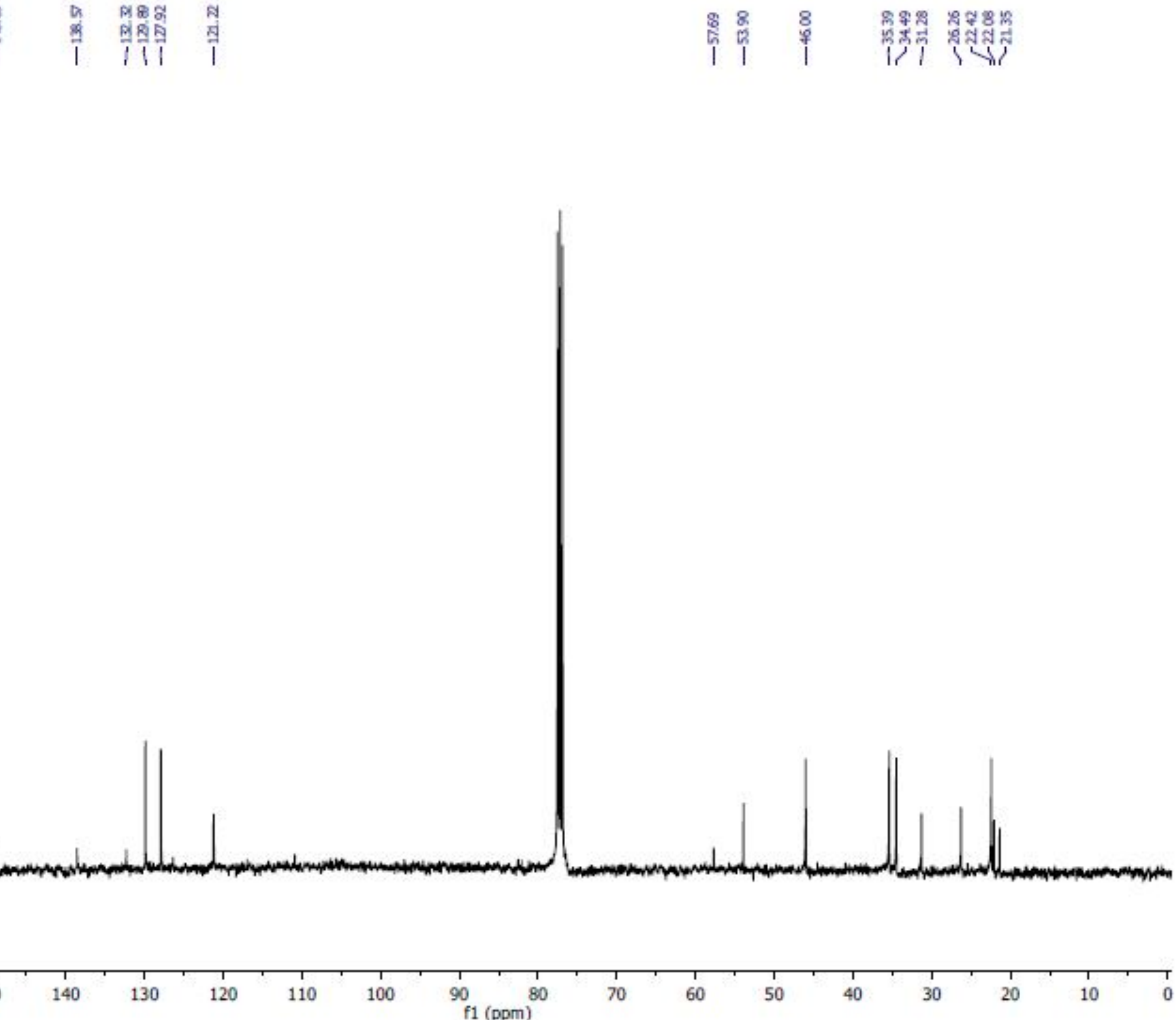
148.89
138.57
132.32
129.89
127.92
121.22
57.69
53.90
46.00
35.39
34.49
31.28
26.26
22.42
22.08
21.35
170
160
150
140
130
120
110
100
90
80
70
60
50
40
30
20
10
0
f1 (ppm)

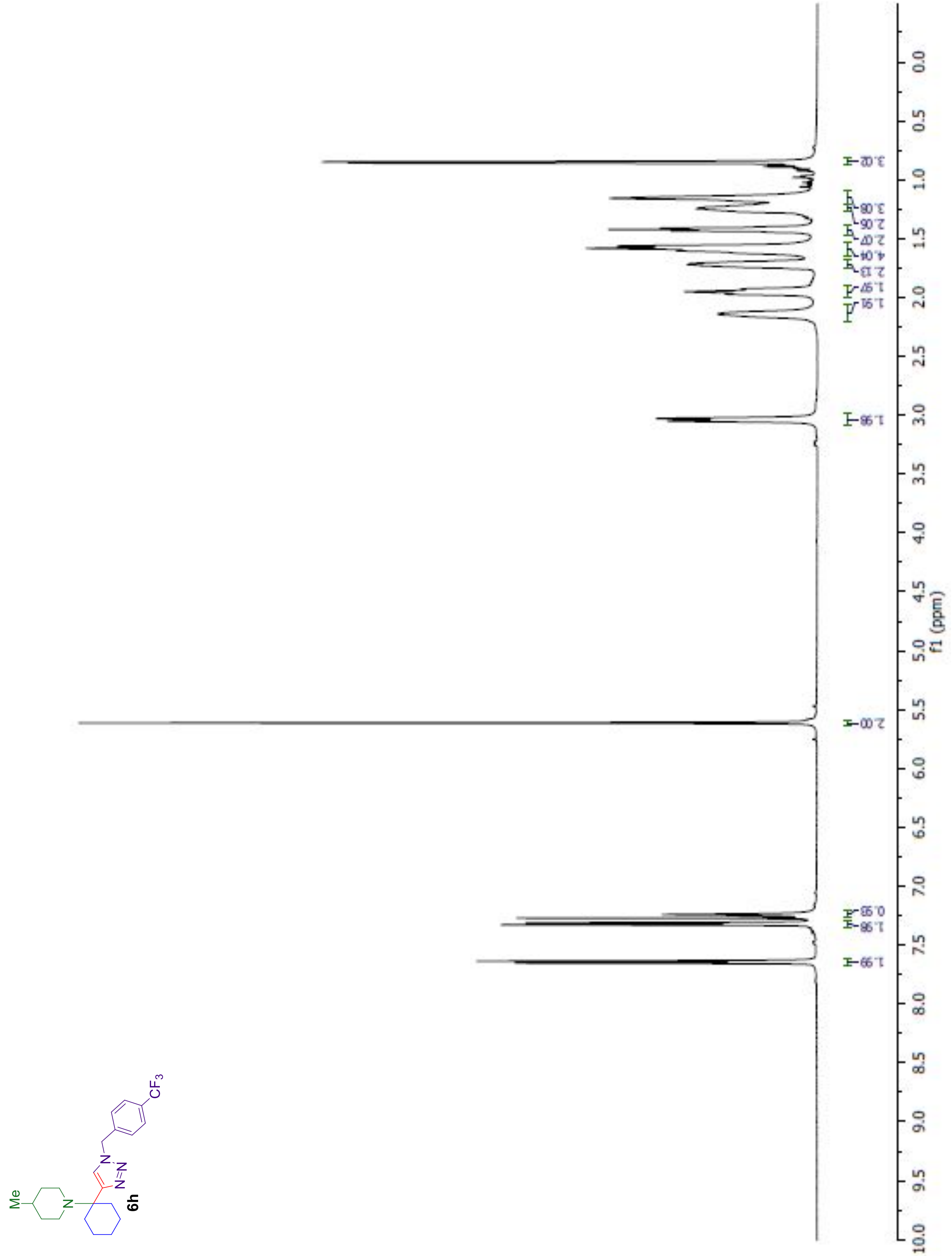
Me
N
N
N=N
CF3
6h
3.08
3.08
2.06
2.07
4.04
2.13
1.97
1.91
1.98
2.00
0.98
1.98
1.99
f1 (ppm)

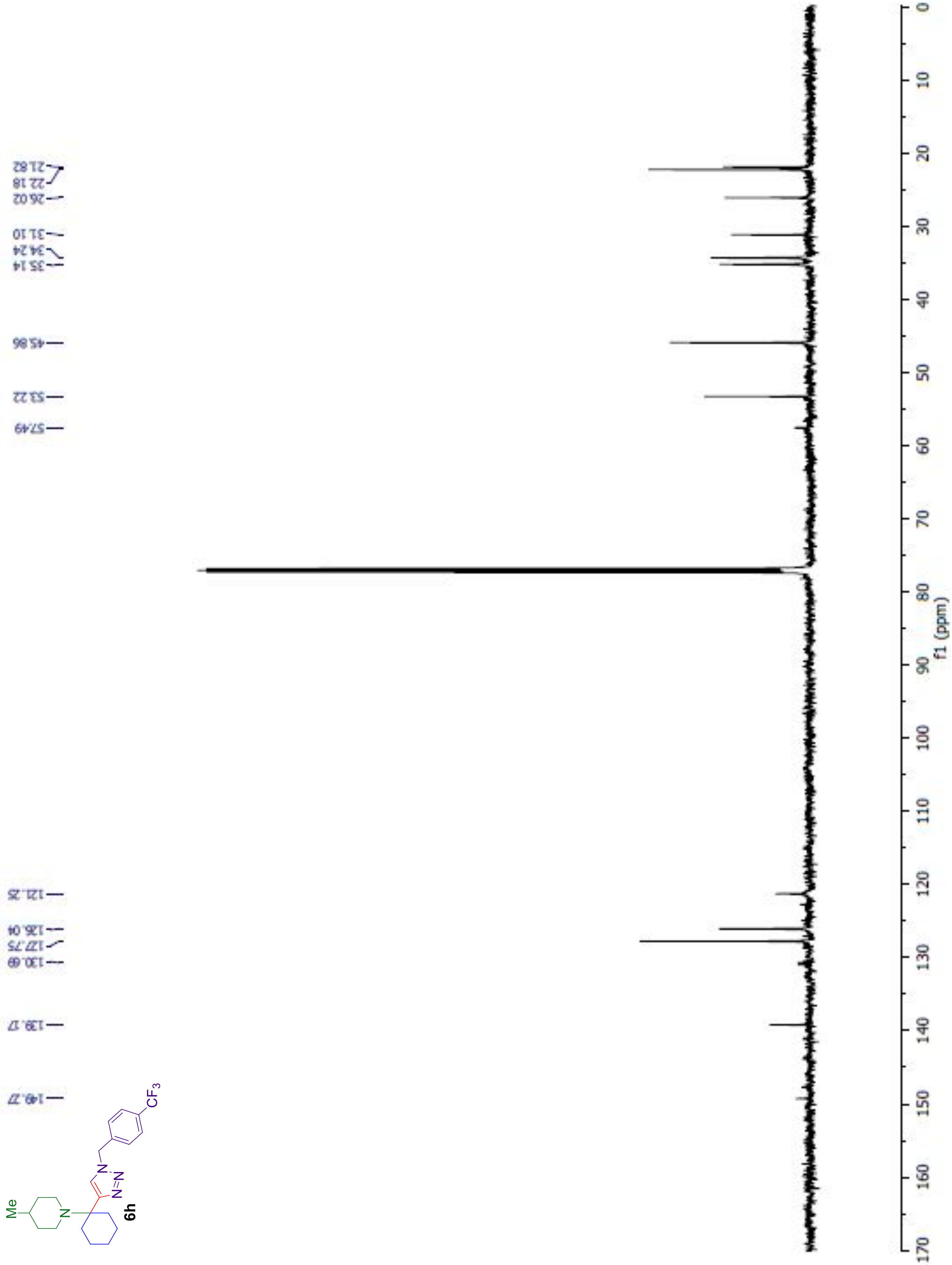
21.82
22.18
26.02
31.10
34.24
35.14
45.86
53.22
57.49
121.25
126.04
127.75
130.69
139.17
149.27
Me
N
N
N=N
CF3
6h
f1 (ppm)
0 10 20 30 40 50 60 70 80 90 100 110 120 130 140 150 160 170

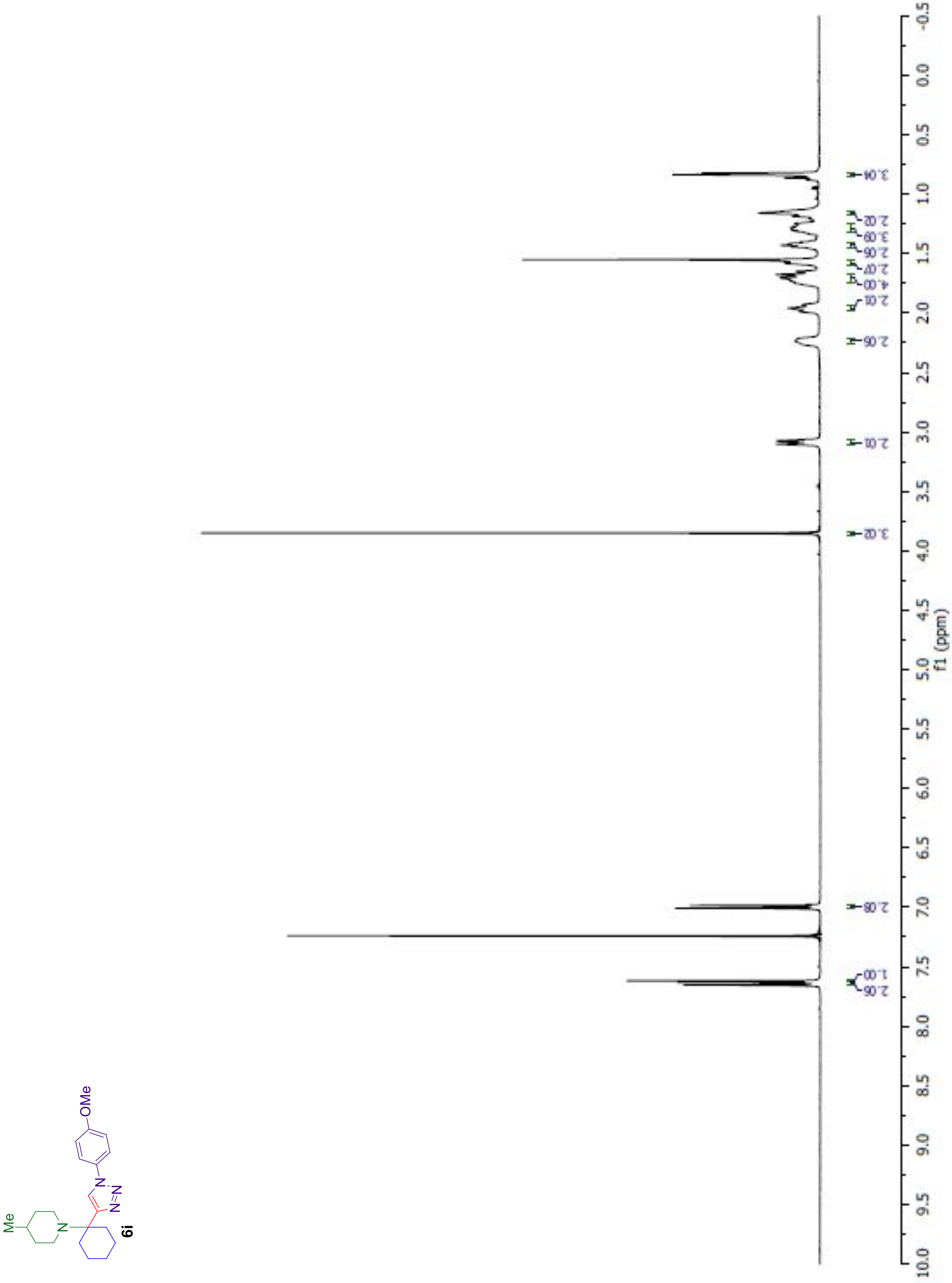
Me
N
OMe
N
N=N
f1 (ppm)
10.0
9.5
9.0
8.5
8.0
7.5
7.0
6.5
6.0
5.5
5.0
4.5
4.0
3.5
3.0
2.5
2.0
1.5
1.0
0.5
0.0
-0.5

6i

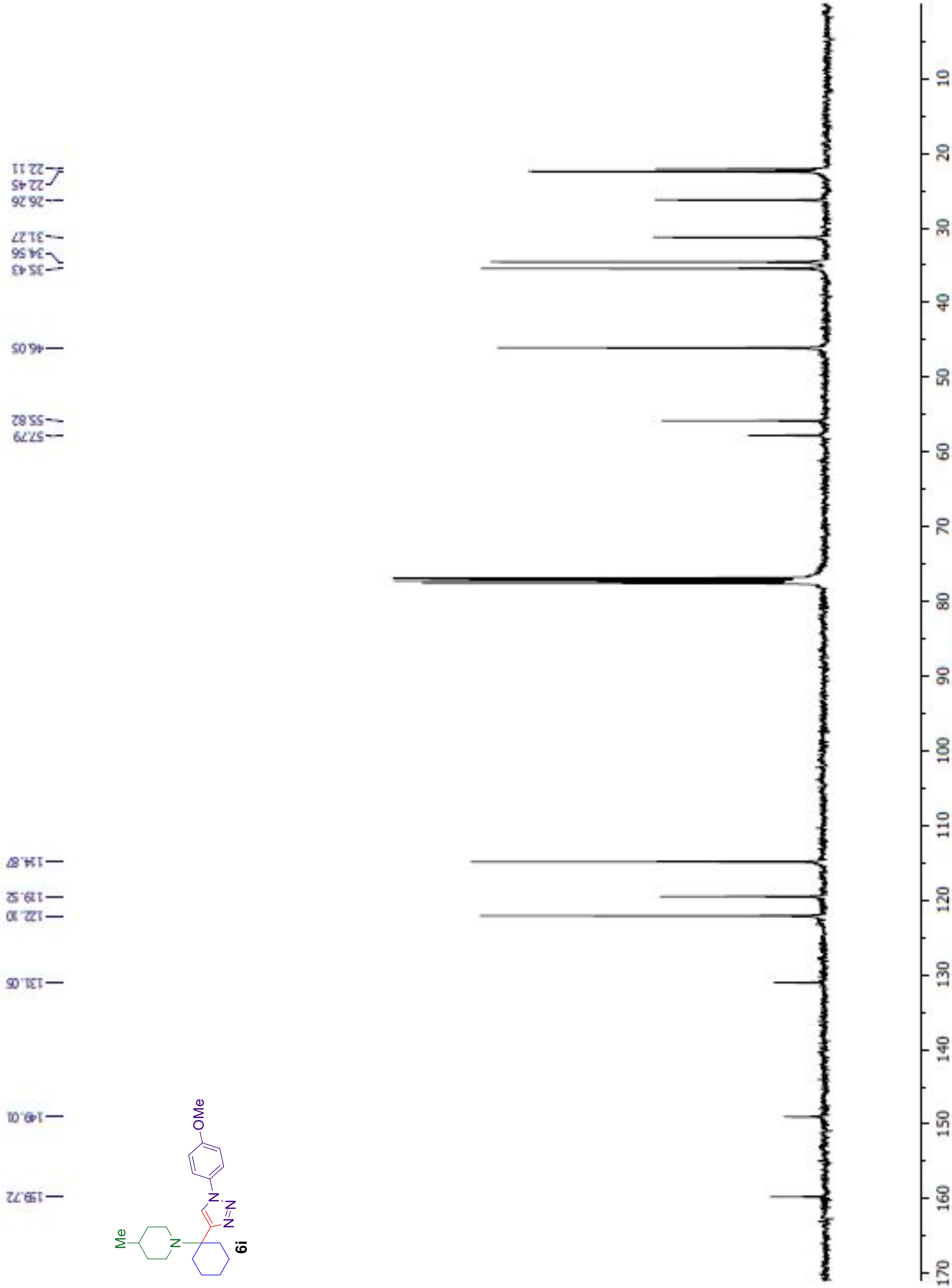
22.11
22.45
26.26
31.27
34.56
35.43
46.05
55.82
57.79
114.87
119.52
122.10
131.05
149.01
159.72
OMe
N
N=N
Me
N
6i
f1 (ppm)
10 20 30 40 50 60 70 80 90 100 110 120 130 140 150 160 170

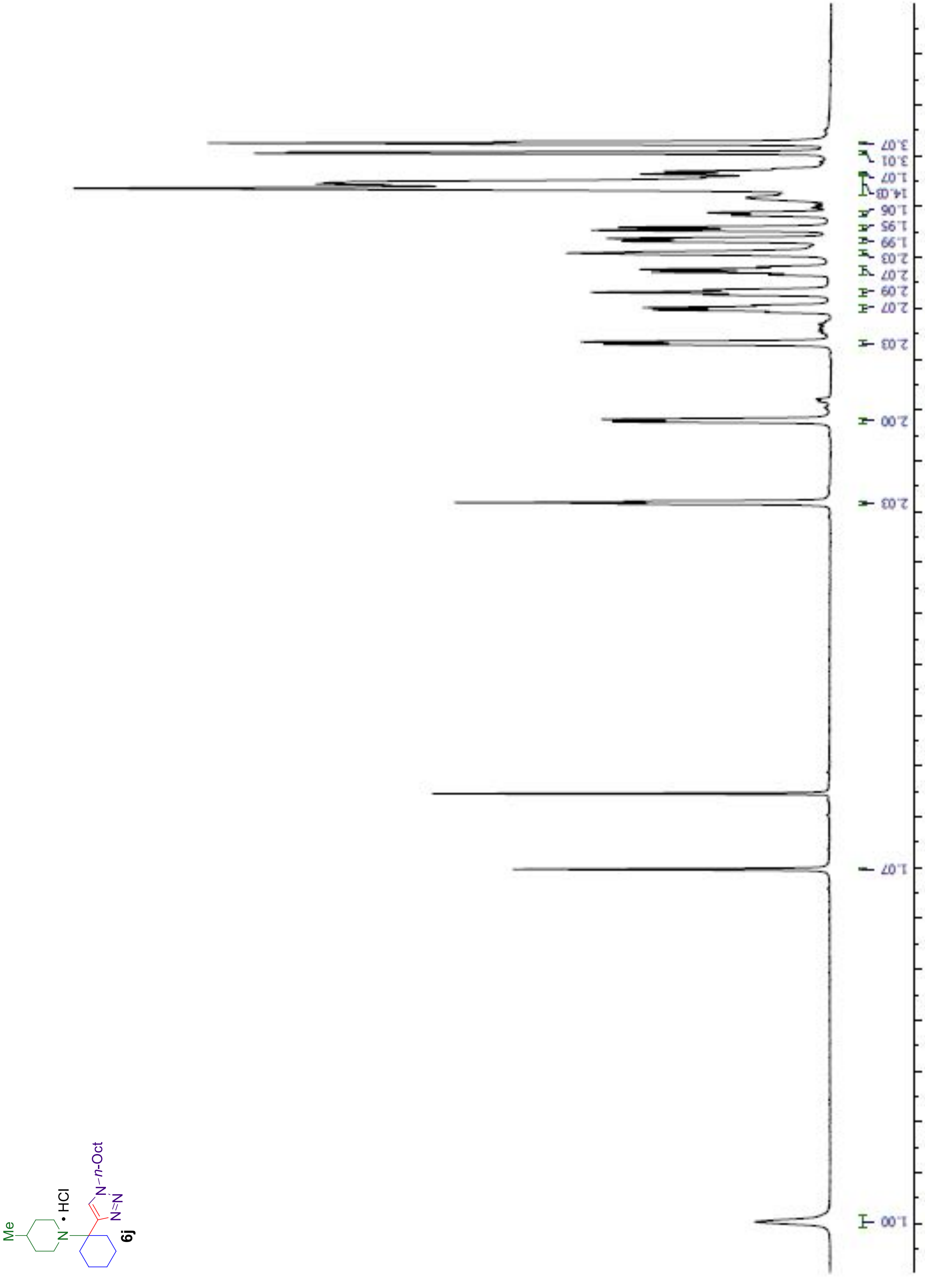

Me
N • HCl
N–n-Oct
N=N
6j
f1 (ppm)

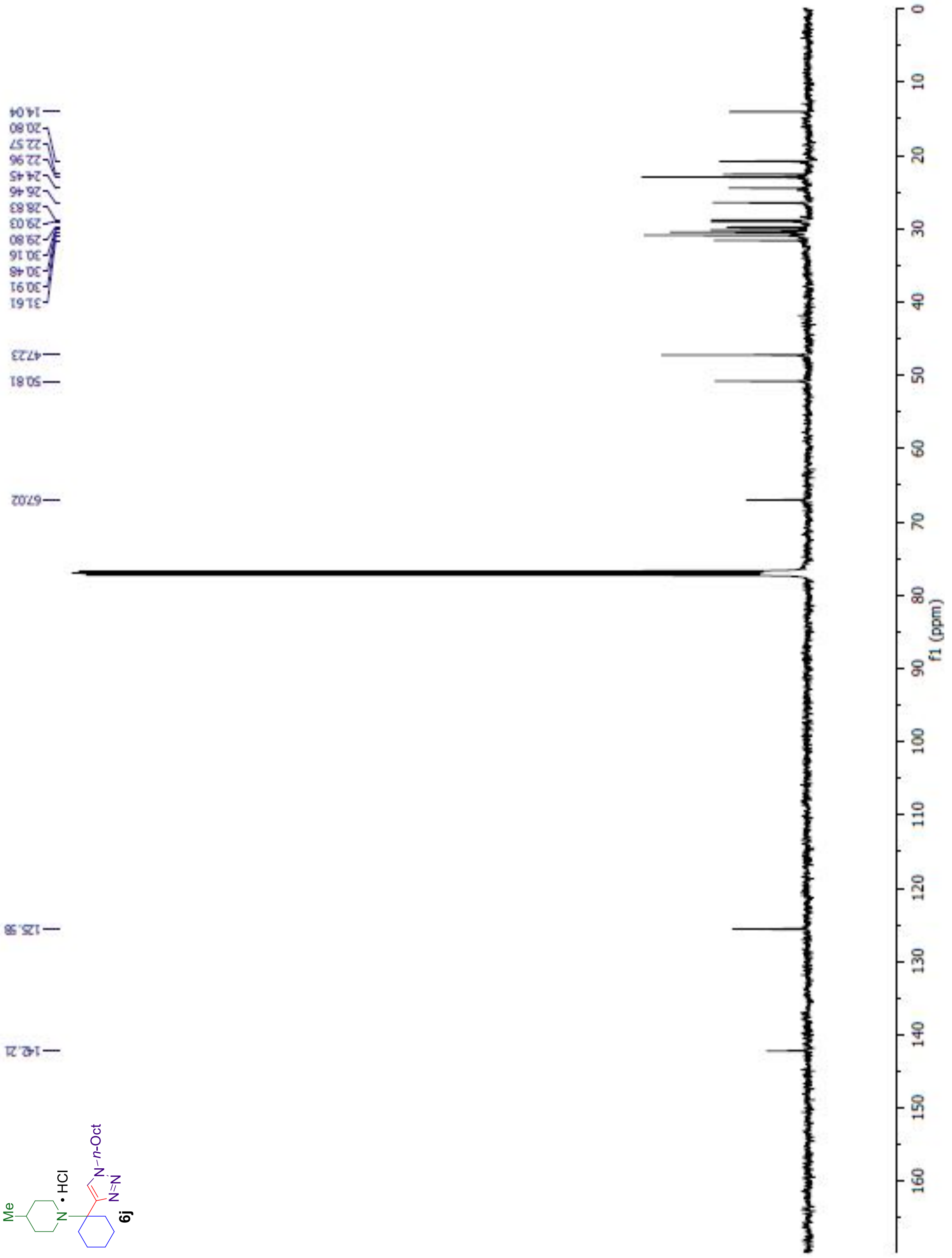
14.04
20.80
22.57
22.96
24.45
26.46
28.83
29.03
29.80
30.16
30.48
30.91
31.61
47.23
50.81
67.02
125.58
142.21
Me
N
• HCl
N–n-Oct
N=N
6j
f1 (ppm)
